# Restoration of energy homeostasis by SIRT6 extends healthy lifespan

**DOI:** 10.1038/s41467-021-23545-7

**Published:** 2021-05-28

**Authors:** A. Roichman, S. Elhanati, M. A. Aon, I. Abramovich, A. Di Francesco, Y. Shahar, M. Y. Avivi, M. Shurgi, A. Rubinstein, Y. Wiesner, A. Shuchami, Z. Petrover, I. Lebenthal-Loinger, O. Yaron, A. Lyashkov, C. Ubaida-Mohien, Y. Kanfi, B. Lerrer, P. J. Fernández-Marcos, M. Serrano, E. Gottlieb, R. de Cabo, H. Y. Cohen

**Affiliations:** 1 The Mina & Everard Goodman Faculty of Life Sciences, Bar-Ilan University, Ramat-Gan, Israel; 2 Translational Gerontology Branch, Intramural Research Program, National Institute on Aging, NIH, Baltimore, MD USA; 3 Technion Integrated Cancer Center, Faculty of Medicine, Technion (Israel Institute of Technology), Haifa, Israel; 4 Metabolic Syndrome Group–BIOPROMET, Madrid Institute for Advanced Studies-IMDEA Food, CEI UAM + CSIC, Madrid, Spain; 5 Institute for Research in Biomedicine (IRB Barcelona), Barcelona Institute of Science and Technology (BIST), Catalan Institution for Research and Advanced Studies (ICREA), Barcelona, Spain

**Keywords:** Ageing, Medical research

## Abstract

Aging leads to a gradual decline in physical activity and disrupted energy homeostasis. The NAD^+^-dependent SIRT6 deacylase regulates aging and metabolism through mechanisms that largely remain unknown. Here, we show that SIRT6 overexpression leads to a reduction in frailty and lifespan extension in both male and female B6 mice. A combination of physiological assays, in vivo multi-omics analyses and ^13^C lactate tracing identified an age-dependent decline in glucose homeostasis and hepatic glucose output in wild type mice. In contrast, aged SIRT6-transgenic mice preserve hepatic glucose output and glucose homeostasis through an improvement in the utilization of two major gluconeogenic precursors, lactate and glycerol. To mediate these changes, mechanistically, SIRT6 increases hepatic gluconeogenic gene expression, de novo NAD^+^ synthesis, and systemically enhances glycerol release from adipose tissue. These findings show that SIRT6 optimizes energy homeostasis in old age to delay frailty and preserve healthy aging.

## Abstract (web summary)

Aging is associated with increased frailty and disrupted energy homeostasis. Here, the authors show that SIRT6 overexpression extends the lifespan of male and female mice and demonstrate that SIRT6 optimizes energy homeostasis in old age, which delays frailty and preserves healthy aging.

## Introduction

Aging is associated with an overall decline in health, increased frailty and is a major risk factor for multiple chronic diseases^1^. Therefore, increasing our understanding of the mechanisms underlying aging processes is a top priority to enable the development of interventions that will lead to the preservation of health and improvements on survival/lifespan.

A growing body of evidence indicates that diet and metabolism are key targetable regulators of healthy lifespan^2^. Dietary restriction (DR), a reduced calorie intake without malnutrition (calorie restriction, CR), as well as some fasting regimens, provide profound health benefits and lead to lifespan extension^3–5^. Similarly, pharmacological or genetic inhibition of the major nutrient-abundance-sensing signaling pathways, mammalian target of rapamycin (mTOR) and insulin/IGF-1, were shown to improve lifespan in organisms ranging from yeast to mammals^6^. Likewise, activation of pathways which sense a low-energy state, such as AMP kinase (AMPK) and sirtuins lead to increased longevity in a wide range of organisms^6^.

Previous studies have shown a loss of metabolic homeostasis with aging. At the level of the whole organism, aged mice and humans show changes in energy expenditure and metabolic flexibility^7,8^. At the cellular level, aging affects various metabolic pathways, many of which are associated with a decline in mitochondrial function^8,9^. Notably, the levels of key metabolites that are consumed by cells for energy production, such as glucose, amino acids (AAs), and lipids, are altered with age in the circulation and in tissues^7,10^. Yet, despite such extensive studies, a comprehensive description of age-related metabolic alterations is lacking. Moreover, it is still unclear why the ability to maintain energy homeostasis is lost with age. The age-dependent changes in metabolite abundance together with the decline in mitochondrial function^9^, suggest a global decrease in energy production with age. Interestingly, many of the aforementioned interventions that promote longevity also activate mitochondrial function and energy metabolism^11^, suggesting that enhancing energy production may be beneficial for extending healthy lifespan.

An essential metabolic process for providing energy to the body is gluconeogenesis (GNG). GNG, the de novo synthesis of the body’s primary source of fuel, glucose, from non-carbohydrate precursors, occurs mainly in the liver, and to a lesser extent in the kidney and gastrointestinal tract. This process is necessary for maintaining blood glucose during fasting and physical activity, and contributes ~64% of total glucose production even during the first 22 h of fasting in humans^12^. GNG is regulated through complex pathways, which include extra- and intra-hepatic mechanisms^13^. Given the major role of GNG in energy production, one would expect GNG to play a major role in aging and frailty. Yet, the effect of age on GNG capacity in mammals is unclear, and reported studies were mostly performed in cell culture. While some studies showed an increase in GNG capacity with age^14^, others reported a decrease^15–17^. These contradictory findings can stem from different experimental systems or gluconeogenic precursors used. Thus, an extensive examination of the effect of aging on energy production and GNG, and its potential connection to age-associated frailty, is required.

Sirtuins are nicotinamide adenine dinucleotide (NAD^+^)-dependent protein deacylases and mono-ADP-ribosyl transferases, which are highly conserved from yeast to mammals. Sirtuins were implicated in many cellular pathways, including DNA repair, metabolism, inflammation, cancer, and aging^18^. Of the seven mammalian sirtuins, SIRT1-7, SIRT1, and SIRT6 protein levels increase upon dietary restriction and fasting in various mouse tissues and human cell lines^19–21^. While most SIRT1 knockout (KO) mice die perinatally, in a few weeks age^22,23^, 129svJ background SIRT6 KO mice exhibit severe developmental defects but survive to about 4 weeks of age^24^. Similarly, in humans and primates, mutations resulting in SIRT6 inactivation result in prenatal or perinatal lethality accompanied by severe developmental brain defects^25^. Interestingly, whole-body SIRT1 overexpression in mice leads to improvement in parameters reflecting healthspan, but not lifespan^26^. Whereas whole-brain-specific SIRT1 overexpression did not affect lifespan and brain plasticity, hypothalamic SIRT1 overexpression delays aging^27,28^. However, whole-body SIRT6 overexpression in the mixed-CB6 mouse background leads to a significant extension of male lifespan and healthspan, associated with inhibition of IGF-1 signaling^29,30^.

A significant amount of data demonstrate the major role of SIRT6 in metabolism^31^. SIRT6 represses glycolysis in an HIF1α-dependent manner^32^, thereby acting as a tumor suppressor by inhibiting the Warburg effect^33^. Liver-specific deletion of SIRT6 results in increased glycolysis, triglyceride synthesis, reduced β-oxidation, and fatty liver formation^34^. Similarly, SIRT6 heterozygotic mice show significantly reduced PPARα-induced β-oxidation^35^. Liver-specific SIRT6 induction showed that SIRT6 negatively regulates GNG by regulating PGC1α and FOXO1 activities^36,37^. However, Deng and his colleagues^34^ showed no effect of liver-specific SIRT6 KO on GNG. Importantly, these metabolic roles of SIRT6 were determined in young mice. Thus, given SIRT6’s major role in aging, the broader metabolic role of SIRT6 under fasting as well as the effect of SIRT6 overexpression on metabolism in the context of aging should be described.

Here, we show that overexpression of SIRT6, but not SIRT1, extends lifespan in C57BL/6JOlaHsd mice in both sexes. Overexpression of SIRT6 reduced the age-related metabolic decline in energy metabolism pathways and inhibited frailty by preserving hepatic NAD^+^ levels, GNG capacity, and maintenance of normoglycemia, key markers of healthy aging. These results emphasize the potential of targeting SIRT6 for maintaining energy metabolism and reducing age-related frailty.

## Results

### SIRT6, but not SIRT1, regulates lifespan in C57BL/6JOlaHsd mice in both sexes

**Fig. 1:**
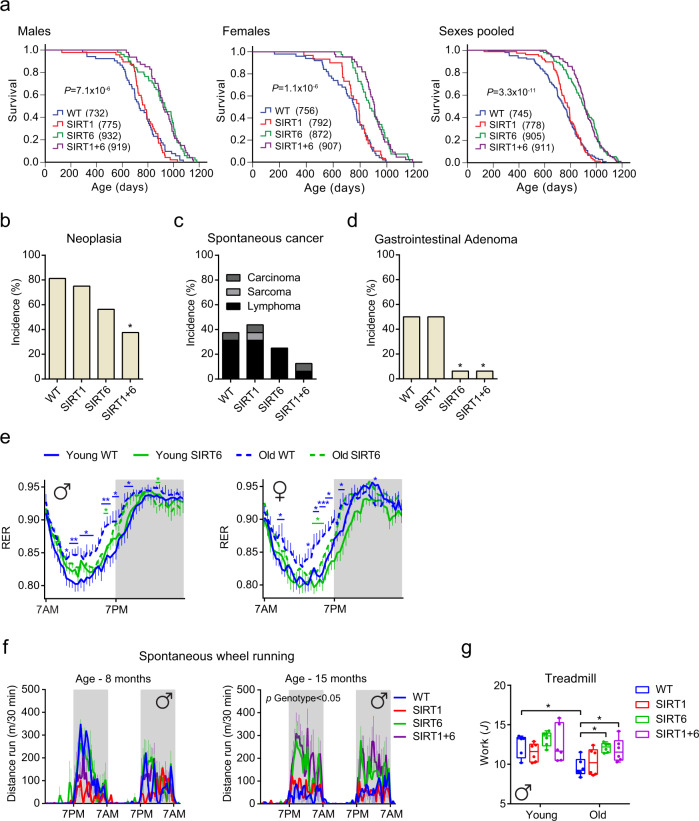
SIRT6 regulates lifespan and healthspan of C57BL/6JOlaHsd mice of both sexes. **a** Kaplan–Meier survival curves for male, female, and combined sexes of WT (*n* = 52 males, *n* = 50 females), SIRT1-tg (*n* = 47 males, *n* = 30 females), SIRT6-tg (*n* = 51 males, *n* = 41 females), and SIRT1 + 6-tg (*n* = 47 males, *n* = 44 females) mice. *p*-values for SIRT6-tg vs. WT mice are shown and were derived from two-tailed log-rank calculations. Median lifespan for each genotype is shown in parentheses. **b**–**d** Neoplasia (**b**), cancer (**c**), and gastrointestinal adenoma (**d**) incidence at the age of 25 months. For (**b**–**d**), *n* = 16 mice per genotype, a mixture of both sexes. Two-tailed Fisher’s exact test; *p < 0.05 vs. WT. Exact *p*-values are reported in Supplementary Table 1. **e** Respiratory exchange ratio (RER) averaged over 3 days in young and old WT and SIRT6-tg mice from either sex measured every half-hour. Three-way ANOVA with repeated measures with age, genotype, and time as variables. Sidak’s post hoc; young WT (* in blue) or old SIRT6 (* in green) vs. old WT. *n* = 8 mice per group, except for young SIRT6 where *n* = 7 mice. **f** Spontaneous wheel running activity of males at the ages of 8 months (*n* = 8 mice per genotype) and 15 months (*n* = 9 mice for WT, SIRT1, SIRT6, *n* = 7 mice for SIRT1 + 6). Two-way ANOVA with time and genotype as variables. **g** Exercise ability on treadmill in young and old males. Two-way ANOVA with Sidak’s post hoc. *n* = 6 mice per group, except for young WT where *n* = 5 mice. *p < 0.05, **p < 0.01, ***p < 0.001. In (**e**) and (**f**), values are shown as mean ± SEM. In (**g**), box extends from the 25th to 75th percentiles, line in the middle of the box is the median and whiskers go down to the smallest value and up to the largest. For (**e**–**g**), exact *p*-values are reported in the Source Data file.

To explore the role of SIRT6 and its interaction with SIRT1 in aging, the lifespan of SIRT1-, SIRT6-, and SIRT1 + SIRT6-overexpressing C57BL/6JOlaHsd transgenic (tg) male and female mice were compared to their wild-type (WT) littermates. Previously we showed that SIRT6 overexpression in mixed-CB6 background extend only male lifespan^29^. As depicted in Fig. 1a, SIRT6 overexpression in C57BL mice led to a 27% and 15% extension in median lifespan, in males and females, respectively (*p* = 7.1 × 10^−6^ and 1.1 × 10^−6^). Likewise, in comparison to their WT littermates, SIRT6 overexpression induced a 11% and 15% extension in maximal lifespan in males and females, respectively (*p* = 0.007 and 0.001). Similarly, SIRT1 + 6-tg mice exhibited a 25% and 20% extension in median lifespan (*p* = 1.1 × 10^−6^ and 1.2 × 10^−8^), and 13% and 15% extension in maximal lifespan (*p* = 0.01 and 0.001), in males and females, respectively. Relative to WT littermates, overexpression of SIRT1 alone did not affect median or maximal lifespan, in accordance with previous data^26^. However, SIRT1-tg mice did show improved survival when comparing the 20th percentile in transgenic versus WT (*p* = 0.01 on pooled sexes). Thus, although SIRT1 may have some beneficial effects on early-age healthspan, SIRT6, but not SIRT1, regulates lifespan in C57BL/6JOlaHsd mice independent of sex, and SIRT1 does not synergize with SIRT6 to further increase median or maximal survival.

### SIRT6 improves healthspan in aged mice

We therefore continued our study of SIRT6, to determine the mechanism(s) for the effects on lifespan. SIRT6 possesses tumor suppressor activity^33^. Thus, we first sought to assess whether this activity contributes to the prolonged lifespan of SIRT6 and SIRT1 + 6 genotypes. At 25 months of age, SIRT1 + 6-tg mice exhibited significantly fewer neoplasms (Fig. 1b). No difference in neoplasm incidence was observed at time of natural death, indicating that SIRT1 + 6-tg mice develop neoplasms significantly later at life, but ultimately die with similar neoplastic load. A similar trend was observed in SIRT6-tg mice (Fig. 1b and Supplementary Fig. 1a). Cancer incidence was similar between genotypes at the time of natural death, and non-significantly lower in SIRT6 and SIRT1 + 6-tg mice at 25 months of age (Fig.1c and Supplementary Fig. 1b). Nevertheless, the prevalence of gastrointestinal adenomas was significantly lower in SIRT6-tg and SIRT1 + 6-tg mice both at 25 months of age and at natural death (Fig. 1d and Supplementary Fig. 1c). Most other pathologies analyzed did not differ between genotypes, although some inflammatory and degenerative processes were significantly less common in the transgenic mice (see Supplementary Table 1). Collectively, as previously suggested^29^, autopsy results indicate that cancer onset might be delayed in long-lived SIRT6-tg and SIRT1 + 6-tg mice.

Next, we determined various healthspan parameters in mice of different age groups. No significant difference in weight was found between various genotypes, up to 1 year of age. After the age of 1 year, in comparison to WT littermates, SIRT6-tg and SIRT1 + 6-tg mice were slightly heavier (4–7% increase), independent of sex (Supplementary Fig. 1d), with increased fat percentage in SIRT6-tg mice (Supplementary Fig. 1e). Importantly, female SIRT6-tg and SIRT1 + 6-tg preserved body weight in advanced age, which is independently associated with increased survival in mice and humans^38,39^ (Supplementary Fig. 1d, f).

In mice and humans, anemia is a common age-related chronic condition^40,41^. Aged WT mice had lower red blood cell (RBC) count, hematocrit (Hct), hemoglobin (Hb), and mean corpuscular hemoglobin concentration (MCHC). Interestingly, all these parameters remained higher in old SIRT6-tg mice, at levels similar to those in young WT mice (Supplementary Fig. 1g and Supplementary Table 2). Moreover, platelet count significantly increased in aged mice, but was maintained at low levels in old SIRT6-tg mice (Supplementary Fig. 1g). Blood chemistry analyses showed that LDL/HDL ratio increased with age, yet remained low in SIRT6-overexpressing animals (Supplementary Fig. 1h). In addition, serum albumin, which is essential for maintenance of blood osmotic pressure and as a carrier for various blood factors and nutrients such as fatty acids, was significantly higher in SIRT6-tg compared to WT independent of age (Supplementary Fig. 1h). Finally, serum IGF-1 levels, lower levels of which are associated with increased lifespan, were lower in SIRT6 and SIRT1 + 6-tg, but not in SIRT1-tg mice, independent of sex (Supplementary Fig. 1i). Other blood parameters analyzed did not differ between genotypes (Supplementary Tables 2 and 3). Altogether, SIRT6 overexpression significantly improved a large set of blood healthspan markers.

Metabolic dysfunction is a hallmark of aging^42^. Thus, whole-body in vivo metabolism was followed in young and old mice using metabolic cages. Food intake increased with age in females but was similar between genotypes irrespective of age (Supplementary Fig. 2a). In comparison to old mice, young mice showed higher metabolic flexibility, seen by circadian fuel source switch from carbohydrate to fatty acids, as measured by the respiratory exchange ratio (RER). Interestingly, old SIRT6-tg mice maintained a young-like oscillatory RER pattern (Fig. 1e and Supplementary Fig. 2b). The change in RER was primarily seen during the light-phase, indicating that SIRT6 overexpression overcomes an age-dependent defect in daily transition from carbohydrate to fat oxidation. O_2_ consumption and CO_2_ production (normalized to body weight) decreased with age in males, but not in females, with no effect of genotype (Supplementary Fig. 2c, d).

Physical activity is a main criterion in the frailty index of aged mice and is clinically relevant to human aging^43^. Thus, selected physical-activity parameters were compared between genotypes, including in-cage locomotor, rearing activities, and spontaneous wheel and forced treadmill running. With age, WT male mice showed unchanged locomotor and rearing activities, but a significant reduction in spontaneous wheel and forced treadmill running (Fig. 1f, g and Supplementary Fig. 2e–g, i). Female mice showed an age-dependent reduction in running wheel activity (Supplementary Fig. 2g). Strikingly, in males, SIRT6 overexpression repressed this age-dependent decline in physical activity. In comparison to 15 months old WT male littermates, SIRT6-tg and SIRT1 + 6-tg male mice ran significantly longer distances and spent more voluntary time on the running wheel (Fig. 1f and Supplementary Fig. 2h). SIRT1 + 6-tg average and maximal running speeds were significantly higher (Supplementary Fig. 2h). At the older age of 22 months, the wheel activity of SIRT6 and SIRT1 + 6-tg mice was still maintained higher than WT littermates (Supplementary Fig. 2g). SIRT6 overexpression in male mice also protected against the age-related decline in treadmill performance, as running duration and distance traveled, and overall amount of work performed were significantly higher in old SIRT6-tg and SIRT1 + 6-tg mice relative to old WT littermates (Fig. 1g and Supplementary Fig. 2i). No difference in treadmill performance was found between SIRT1-tg mice and WT littermates. Young female SIRT6-tg mice showed a non-significant increase in in-cage locomotor and rearing activities, with no change in spontaneous wheel or forced treadmill running at old age (Supplementary Fig. 2e–g, j). Taken together, overexpression of SIRT6 alone or with SIRT1 significantly improves healthspan, promotes physical activity performance, and reduces frailty at old age.

### SIRT6 affects GNG and tricarboxylic acid (TCA) cycle-related serum metabolites during fasting

**Fig. 2:**
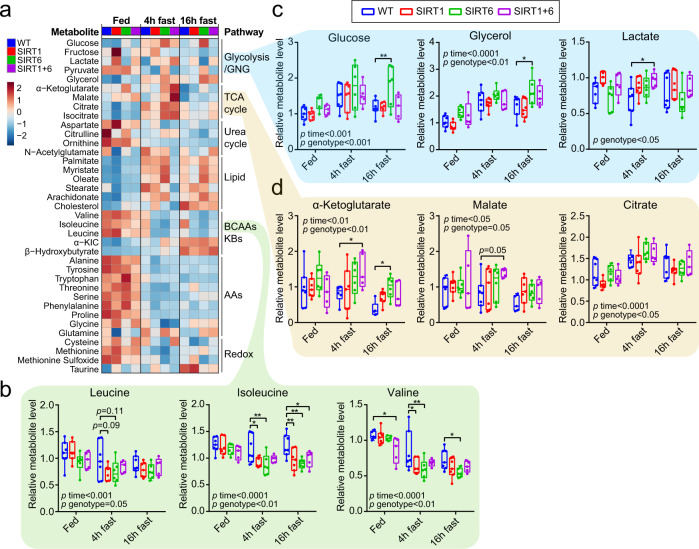
Serum metabolites associated with fasting and sirtuin overexpression. **a** Heatmap showing serum metabolites from key metabolic pathways that changed significantly either with dietary state (fed, and 4 h and 16 h of fasting), genotype, or both as measured in two-way ANOVA. Each square represents average metabolite abundance of 7 WT mice, 7 SIRT1-tg mice, 6 SIRT6-tg mice, and 5 SIRT1 + 6-tg mice. **b**–**d** Scatterplots of data from (**a**) showing the levels of BCAAs (**b**), glucose and the GNG precursors glycerol and lactate (**c**), and TCA cycle metabolites (**d**). For (**b**–**d**), *n* = 7 WT mice, *n* = 7 SIRT1-tg mice, *n* = 6 SIRT6-tg mice, and *n* = 5 SIRT1 + 6-tg mice. Box extends from the 25th to 75th percentiles, line in the middle of the box is the median and whiskers go down to the smallest value and up to the largest. Statistical analysis was performed using two-way ANOVA (time, genotype) with Dunnett’s post hoc correction. *p*-value for genotype or fasting effect is indicated in graphs, exact *p*-values are reported in Supplementary Table 4. *p < 0.05, **p < 0.01.

Surprisingly, despite the extensive research on the metabolic roles of SIRT1 and SIRT6, little is known about their impact at the in vivo metabolite levels. Thus, serum metabolomics was performed in 15 months old mice from the four genotypes in the fed state, and after 4 and 16 h fasting. Principal component analysis (PCA) of all identified metabolites showed that fasting profoundly shifted the serum metabolome as early as after 4 h (Supplementary Fig. 3a). Notably, fasting increased the levels of circulating free fatty acids (FFAs) and β-hydroxybutyrate, likely due to activation of lipolysis and ketogenesis. In contrast, multiple circulating AAs, including the branched chain amino acids (BCAAs) isoleucine and valine, were depleted upon fasting, likely due to depletion of dietary AAs (Fig. 2a and Supplementary Fig. 3b, c; see also Supplementary Table 4 for source data). During fasting, levels of these BCAAs were significantly lower in the mice overexpressing SIRT1, SIRT6, or both (Fig. 2b). WT mice show a reduction in serum glucose levels between 4 and 16 h of fasting. Interestingly, SIRT6, but not SIRT1, overexpression suppressed this response (Fig. 2c). These findings indicate that compared to WT mice, SIRT6-tg mice are more efficient in maintaining blood glucose levels during fasting.

Maintaining higher glucose levels during fasting in SIRT6-tg suggest higher GNG in these mice. Glycerol, lactate, pyruvate, and alanine are the main gluconeogenic substrates. SIRT6 overexpression led to significantly higher serum glycerol levels at 16 h fast, which correlated positively with serum glucose levels (Fig. 2c and Supplementary Fig. 3e). In contrast, at the age of 15 months, no significant effect for either genotype was found on lactate, pyruvate, and alanine levels upon fasting, with the exception of increased lactate levels in SIRT1 + 6-tg mice at 4 h fast (Fig. 2c and Supplementary Fig. 3d, e). The serum levels of the tricarboxylic acid (TCA) cycle metabolites α-ketoglutarate (αKG), malate, and citrate increased in SIRT6 and SIRT1 + 6-tg, but not in SIRT1-tg, mostly at the 4 h fast (Fig. 2d). Thus, serum metabolomics shows that normoglycemia maintenance and TCA cycle are affected by SIRT6, but not by SIRT1 overexpression.

### SIRT6 restores age-related deterioration in normoglycemia and GNG capacity

**Fig. 3:**
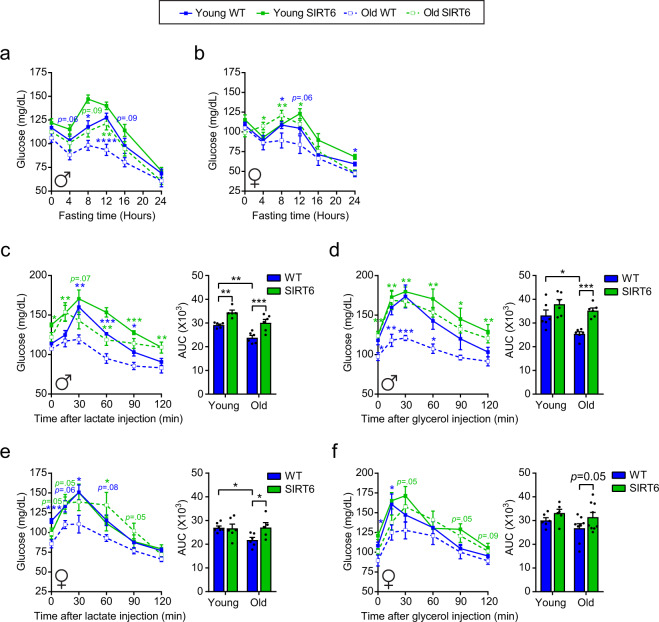
SIRT6 overexpression blocks age-dependent normoglycemia and deterioration in gluconeogenesis capacity. **a** Blood glucose levels in young and old WT (blue) and SIRT6-tg (green) male mice at fed and fasted time points, as indicated in the graph. *n* = 10 young WT mice, *n* = 8 young SIRT6 mice, *n* = 7 old WT and old SIRT6 mice. **b** Same as (**a**), but in females. *n* = 8 young WT mice, *n* = 7 young SIRT6, old WT, and old SIRT6 mice. **c** Lactate tolerance test in 6 h-fasted male mice; *n* = 6 young WT, old WT, and old SIRT6 mice, *n* = 5 young SIRT6 mice. **d** Glycerol tolerance test in 6 h-fasted male mice; *n* = 6 mice per group. **e** Lactate tolerance test in 6 h-fasted female mice; *n* = 6 old WT, young SIRT6, and old SIRT6 mice, *n* = 7 young WT mice. **f** Glycerol tolerance test in 6 h-fasted female mice; *n* = 6 young WT and young SIRT6 mice, *n* = 8 old WT mice, *n* = 9 old SIRT6 mice. The area under the curve (AUC) for each tolerance test is shown on the right. For all panels, ages were 4–6 (young) and 22–24 (old) months. Statistical significance was calculated using three-way ANOVA with Sidak’s post hoc for line graphs, and two-way ANOVA with Fisher’s LSD method for bar graphs. Young WT (* in blue) or old SIRT6 (*in green) vs. old WT. In all panels, values are mean ± SEM. Exact *p*-values are reported in the Source Data file. *p < 0.05, **p < 0.01, ***p < 0.001, ****p < 0.0001.

To further explore the normoglycemic effect of SIRT6, blood glucose levels were measured in young and old, fed or fasted mice after 0, 4, 8, 12, 16, and 24 h of fasting. In young WT mice, a gradual increase in blood glucose levels was found between 4 and 12 h of fasting, followed by a significant decrease until 24 h of fasting. Interestingly, 22 months old WT animals were not able to maintain glucose levels, as blood glucose started to decrease earlier, reaching a significant decrease already after 8 h of fasting (Fig. 3a). Strikingly, glucose levels in old SIRT6-tg mice resembled those of young-WT mice during this fasting period (Fig. 3a). The effect of SIRT6 on preserving normoglycemia during fasting was not sex specific, as similar results were seen in females (Fig. 3b). Additionally, at this age of 22 months, SIRT1 + 6 animals presented the same phenotype as the SIRT6-tg (Supplementary Fig. 4a). Last, compared to young WT, young SIRT6-tg attained higher blood glucose levels. Thus, SIRT6 overexpression blocks the significant age-dependent impairment of normoglycemia and maintains young-like blood glucose levels even at advanced age.

We then hypothesized that SIRT6 may reverse the impaired glucose regulation observed in old mice by activating glycogenolysis and/or gluconeogenesis. After 4 h morning fast, hepatic glycogen levels were similar between genotypes in young mice and higher in old SIRT6-tg mice relative to WT littermates, suggesting net activation of glycogenesis, but not glycogenolysis, in old SIRT6-tg livers (Supplementary Fig. 4b). Consequently, we determined GNG capacity by measuring blood glucose after injection of lactate or glycerol, major gluconeogenic precursors, in young and old mice. In accordance with the glycemic pattern, gluconeogenic capacity from both precursors significantly decreased in old WT male mice but was maintained in old SIRT6-tg male mice at similar levels as in young WT mice (Fig. 3c, d). This effect was independent of sex since similar results were obtained in females (Fig. 3e, f). Likewise, old SIRT1 + 6-tg animals showed a significant increase in GNG capacity from lactate and glycerol at old age (Supplementary Fig. 4c, d). Importantly, glucose tolerance was similar between young and old WT mice and SIRT6-tg mice of both sexes (Supplementary Fig. 4e, f), indicating that the effect could not be attributed to faster glucose clearance in the aged WT mice. Altogether, these results show that SIRT6 overexpression leads to maintenance of young-like GNG in old age due to improved glycerol and lactate utilization under fasting.

### SIRT6 promotes hepatic catabolic and anti-inflammatory pathways

**Fig. 4:**
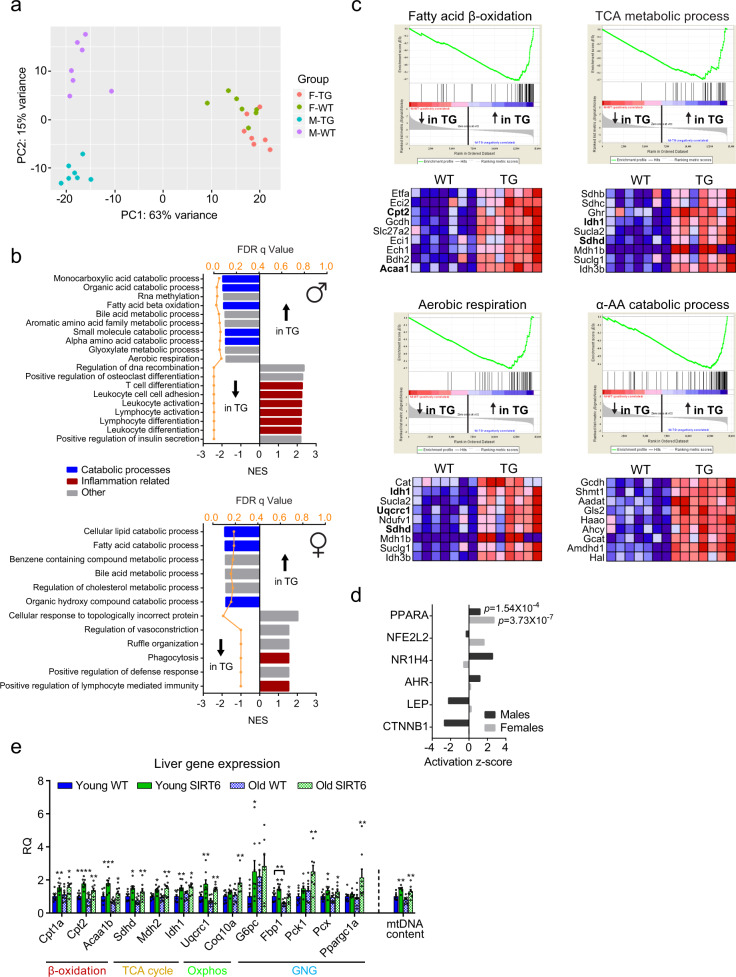
SIRT6 activates hepatic mitochondrial catabolic and anti-inflammatory pathways. RNA-seq was carried out on the liver of 6 months old WT and SIRT6-tg mice from either sex. **a** Principal component analysis was performed on all expressed genes. Each data point represents an individual mouse. **b** Gene Set Enrichment Analysis showing the top pathways up- or down-regulated in SIRT6-tg mice, sorted by normalized enrichment scores (NES). FDR *q*-values are shown in orange. Pathways related to catabolism and to inflammation are presented in blue and red, respectively. **c** Four metabolic pathways up-regulated in livers from male SIRT6-tg mice. In the upper part, each vertical black bar represents a gene in the gene set and its corresponding location in the sorted gene list. In the lower part, corresponding heatmaps show the expression values of the top genes in each pathway, which contribute most to the NES. High expression is colored in red and low expression in blue. Bold text indicates genes validated by qPCR in (**e**). **d** Ingenuity upstream regulator analysis common to males and females, based on genes differentially expressed between WT and SIRT6-tg mice. *p*-values for PPARα are shown, calculated by Fisher’s exact test. **e** Real-time PCR validation of gene expression from the indicated pathways, and mtDNA content in the livers of young and old WT and SIRT6-tg males. Two-way ANOVA with Fisher’s LSD method, asterisks indicate values significantly different between WT and SIRT6-tg at the same age. *n* = 8 mice per group, except for old SIRT6 for *Coq10a* (*n* = 6) and *G6pc* (*n* = 7), and for young WT and young SIRT6 for mtDNA content (*n* = 6). Mice ages are 6 and 25 months. RQ, relative quantity. Bars represent mean ± SEM. Exact *p*-values are reported in the Source Data file. *p < 0.05, **p < 0.01, ***p < 0.001, ****p < 0.0001.

GNG occurs mainly in the liver^44^. To identify hepatic pathways that are associated with the restored gluconeogenic capacity, liver RNA sequencing of 4 h-fasted 6 months old WT and SIRT6-tg male and female mice was performed (Supplementary Table 5). PCA showed that gender was responsible for most of the variance (PC1). In addition, a striking genotype effect was seen in PCA, with the effect of SIRT6 substantially greater in males than females (Fig. 4a). Using Gene Set Enrichment Analysis (GSEA), we found that inflammatory pathways were significantly inhibited in SIRT6-tg males, whereas a similar but milder effect was seen in females (Fig. 4b and Supplementary Table 6). Suggesting that the observed difference in the effects of SIRT6 on lifespan between sexes (Fig. 1a) might stem from its transcriptional regulation.

Metabolism, enriched for catabolic pathways, was strongly up-regulated in both male and female SIRT6-tg mice (Fig. 4b and Supplementary Table 6). Fatty-acid β-oxidation, TCA cycle, aerobic respiration, and AA catabolism gene expression were significantly increased in SIRT6-tg mice (Fig. 4c). These findings suggest that SIRT6 induces fatty acid and AA catabolism in the liver, mimicking the effect of fasting and CR in both mice and primates^44–46^. Analysis of upstream regulators, common to both male and female transcriptomes, showed that PPARα, a nuclear receptor which promotes fatty-acid β-oxidation in the liver^44^ was activated (Fig. 4d). Importantly, the SIRT6-dependent increased expression of key catabolic genes of β-oxidation, TCA cycle, and aerobic respiration, was also maintained at old age (Fig. 4e). In agreement with the effect of SIRT6 on GNG capacity, the expression of the key hepatic GNG genes *Pck1, Pcx*, *G6pc*, and *Fbp1* was higher in SIRT6-tg livers (Fig. 4e). Likewise, the expression of the transcriptional coactivator *Ppargc1a*, which regulates gluconeogenic gene expression and mitochondrial biogenesis, was also increased in old SIRT6-tg. In agreement with this finding, hepatic mitochondrial DNA (mtDNA) content was higher in SIRT6-tg mice (Fig. 4e).

Next, to explore the effect of SIRT6 on protein levels, a quantitative liver proteomics analysis from the same individual male mice utilized for RNA-seq was performed. Liquid chromatography-tandem mass spectrometry (LC-MS/MS) analysis identified and quantified 2785 hepatic proteins. PCA of the proteomics data revealed that groups were clustered by genotype (Supplementary Fig. 5a). The global transcriptome correlated well with the proteome (*r* = 0.59, *p* < 0.0001; Supplementary Fig. 5b). From the total expression list, 185 proteins were differentially expressed between genotypes (Benjamini-Hochberg corrected *p* < 0.05 with ±20% fold change), of which 122 and 63 were down- and up-regulated, respectively, in SIRT6-tg mice (Supplementary Fig. 5c and Supplementary Table 7). In agreement with transcriptomics data, SIRT6 up-regulated proteins were enriched for metabolic-related pathways, whereas down-regulated proteins included immune-related pathways (Supplementary Fig. 5d). Interestingly, proteomics analysis also identified a significant decrease in the immunoproteasome (i-proteasome) regulatory subunits PA28α and PA28β and core subunits β2i and β5i in SIRT6-tg mice. In addition, the β5 constitutive proteasome (c-proteasome) subunit was significantly increased in SIRT6-tg livers (Supplementary Fig. 5e, f). Previous studies showed a role for the i-proteasome in regulating aging. A mouse model with reduced β5 proteasomal activity accumulates ubiquitinated (Ub) proteins and has shortened lifespan^47^, whereas overexpression of β5 in *Drosophila melanogaster* reduces Ub-protein aggregates and increases lifespan^48^. Similarly, we found significantly decreased Ub-protein levels (Supplementary Fig. 5g), suggesting SIRT6 as a key regulator of protein homeostasis (proteostasis). Altogether, hepatic transcriptomic and proteomic profiling revealed the SIRT6-dependent activation of catabolic and anti-inflammatory pathways, which can potentially support the activated GNG (Supplementary Fig. 5h).

### Young-like hepatic TCA cycle and GNG-related metabolite abundance in old SIRT6-tg mice

**Fig. 5:**
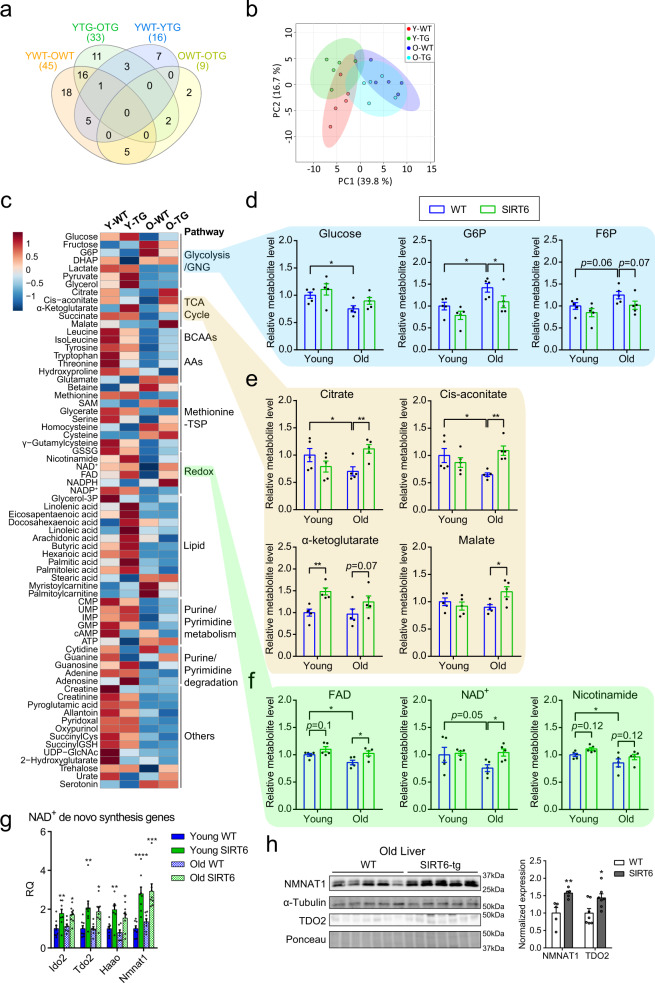
Youthful-like hepatic TCA cycle, GNG, and redox metabolite levels in old SIRT6-tg. **a** Lists of statistically significant metabolites from each of the four comparisons shown were used to create Venn diagram. The number of significant metabolites in each comparison is shown in parentheses. **b** PCA of significantly changed metabolites. Each dot represents one biological replicate. **c** Heatmap of significantly changed metabolites. Each square represents average metabolite abundance of *n* = 5 mice per genotype. TSP, transsulfuration pathway; Y, young; O, old; WT, wild type; TG, transgenic. **d**–**f** Scatterplots showing metabolite levels of hepatic glycolysis/GNG (**d**), TCA cycle (**e**), and redox metabolism (**f**) pathways. *n* = 5 mice per genotype, each dot represents one mouse. **g** Expression of key NAD^+^ de novo synthesis genes in the liver of young and old WT and SIRT6-tg mice. Asterisks indicate values significantly different between WT and SIRT6-tg at the same age. *n* = 8 mice for young WT, young SIRT6, and old SIRT6 (for *Haao* in young SIRT6, *n* = 7); and *n* = 7 mice for old WT (for *Nmnat1*, *n* = 8), males. RQ, relative quantity. **h** Protein levels of NMNAT1 (*n* = 5 mice) and TDO2 (*n* = 7 mice) in livers of old WT and SIRT6-tg littermates. ImageJ quantification of NMNAT1 normalized to α-tubulin and TDO2 normalized to ponceau is shown in the right. For all panels, mice were at the ages of 5–7 months (young) and 20–24 months (old). Bars represent mean ± SEM. For (**d**–**g**) data were analyzed using two-way ANOVA, for (**h**) using two-tailed Student’s *t*-test. Exact *p*-values are reported in the Source Data file. *p < 0.05, **p < 0.01, ***p < 0.001, ****p < 0.0001.

To further understand the liver metabolic profile of SIRT6 in normoglycemia and GNG during aging, liver metabolomics was performed on young and old 6 h-fasted WT and SIRT6-tg mice. Aging had a profound effect on liver metabolite abundance. Out of 125 identified metabolites, 45 were significantly different between young and old WT mice, and 33 between young and old transgenic mice. Genotype also had a significant effect, as 16 metabolites in young animals and 9 metabolites in aged animals significantly differed between WT and SIRT6-tg. Together, a total of 70 significant metabolites were altered by age or genotype (Fig. 5a and Supplementary Table 8). Metabolite Set Enrichment Analysis (MSEA) showed that TCA cycle, mitochondrial electron transport chain, and GNG were affected by age in both WT and transgenic mice. Genotype also affected these three pathways in either young or old mice (Supplementary Fig. 6a). Interestingly, PCA on significant metabolites showed that old SIRT6-tg grouped closer to the younger profile (Fig. 5b). These findings indicate that SIRT6 maintains a global young-like metabolite profile in the liver.

Next, significant metabolites were organized based on their corresponding pathways (Fig. 5c and Supplementary Fig. 6b). Interestingly, similar to blood glucose levels (Fig. 3a), hepatic glucose abundance decreased with age and tended to increase in SIRT6-tg (Fig. 5d). The GNG intermediates glucose-6-phosphate (G6P) and fructose-6-phosphate (F6P) both increased with age, but in aged SIRT6-tg mice, they remained at young-like levels (Fig. 5d). These results are consistent with our findings that SIRT6 blocks the aging-associated decrease in hepatic GNG.

We next examined TCA cycle metabolite levels. SIRT6 overexpression inhibited the age-related decline in the concentrations of citrate and *cis*-aconitate. Additionally, it significantly increased the levels of αKG at young age and malate at old age (Fig. 5e). Of note, citrate activates the key GNG enzyme fructose-1,6-bisphosphatase (FBP1)^49^, suggesting that higher citrate levels can support GNG by activating FBP1. The GNG substrates lactate, pyruvate, and glycerol and numerous AAs that replenish the TCA cycle (Ser, Met, Tyr, Trp, Thr, Leu, and Ile) were depleted in the aged liver (Fig. 5c and Supplementary Fig. 6b–d). In comparison to young WT, young SIRT6-tg has higher pyruvate, glycerol, and poly-unsaturated fatty acid (PUFA) levels. However, old WT and SIRT6-tg have similar levels of these (Fig. 5c and Supplementary Fig. 6b, c, h). Old SIRT6-tg liver shows a trend towards higher AA levels than old WT liver. This elevation might be due to an increased autophagy in old SIRT6-tg mice. Indeed, in comparison to WT liver, SIRT6-tg liver shows a significant increase in autophagy levels as measured by the autophagy markers LC3b-II and p62 (Supplementary Fig. 6i). Importantly, the glucogenic AA serine was significantly higher in old SIRT6-tg liver, similar to the levels found in young WT liver (Supplementary Fig. 6d). These results suggest that TCA cycle and GNG activities decrease in the aged liver due to decreased levels of their precursors, and that SIRT6 might activate these pathways by increasing their precursor levels.

Other metabolites also showed young-like levels in old livers from aged SIRT6-tg mice. The abundance of the major β-oxidation precursors, palmitoyl-carnitine and myristoyl-carnitine increased with aging in old WT mice, but not in old SIRT6-tg mice (Supplementary Fig. 6e). In addition, the disaccharide trehalose, known to extend the lifespan of the nematode *Caenorhabditis elegans*^50^, was depleted in aged WT, but not in aged SIRT6-tg mice (Supplementary Fig. 6f). Likewise, SIRT6-tg mice showed a lower age-dependent reduction in IMP levels (Fig. 5c and Supplementary Fig. 6g). Thus, SIRT6 overexpression inhibits additional aging-related phenomena.

### SIRT6 maintains NAD^+^ levels and increases de novo NAD^+^ synthesis gene expression

Functional redox metabolism, mediated by NAD^+^ and flavin adenine dinucleotide (FAD), is critical for multiple metabolic reactions and is impaired by aging^51^. Interestingly, hepatic NAD^+^ and FAD levels decreased with age. Remarkably, SIRT6 overexpression prevented the age-related decline of NAD^+^ and FAD (Fig. 5f). In addition, nicotinamide (NAM), a product of SIRT6 reaction and an NAD^+^ salvage precursor, also decreased with age, and increased in SIRT6-tg, independently of age (*p* genotype < 0.05; Fig. 5f). The maintained NAD^+^ levels in old SIRT6-tg livers could be due to either reduced consumption or increased production of NAD^+^. The DNA-damage-induced poly [ADP-ribose] polymerase 1 (PARP1) is a major NAD^+^ consumer^52^. Thus, we first tested the possibility that SIRT6 affects NAD^+^ consumption by reducing DNA-damage-induced activation of PARP1. However, we found that hepatic PARP1 levels, global PARylation, and gamma-H2AX levels were similar between genotypes (Supplementary Fig. 6j), suggesting that SIRT6 does enhance NAD^+^ levels by reducing its consumption by PARP1. Next, we sought to determine whether SIRT6 affects NAD^+^ production. Supplementing mice with NAM results in increased expression of de novo NAD^+^ biosynthetic enzymes^53^. In accordance with their higher NAM levels, the hepatic mRNA expression of multiple de novo NAD^+^ biosynthetic genes; *Ido2*, *Tdo2*, *Haao*, and *Nmnat1* was up-regulated in SIRT6-tg (Fig. 5g). In agreement, NMNAT1 and TDO2 protein levels were also significantly increased in old SIRT6-tg livers (Fig. 5h). These findings suggest that SIRT6-tg mice maintain NAD^+^ levels during aging due to increased NAD^+^ production. Collectively, liver metabolomics showed that SIRT6-tg mice present a young-like profile of key metabolites of GNG, TCA cycle, and redox pathways, suggesting that hepatic GNG and TCA cycle activities are increased in SIRT6-tg, at least partially, by augmented NAD^+^ cofactor and glycerol/pyruvate substrate availability.

### SIRT6 restores the aging-associated decline in hepatic lactate oxidation

**Fig. 6:**
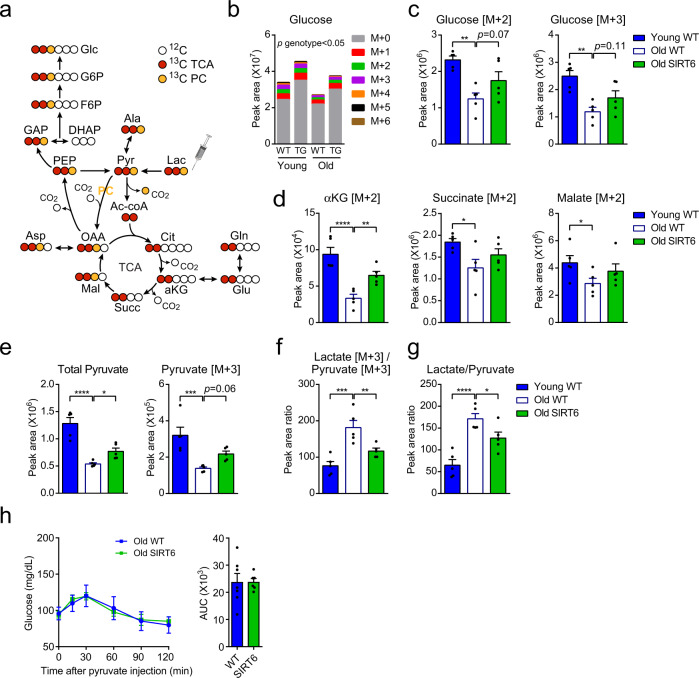
SIRT6 restores the significant reduction in hepatic lactate oxidation and contribution to GNG at old age. **a** Schematic showing fates of lactate carbons following injection of [U-^13^C]-lactate, after one round of the TCA cycle or flux through pyruvate carboxylase (PC, Pyr→OAA). White balls, ^12^C carbons. Colored balls, ^13^C carbons. Red, carbon flux from lactate to glucose via the TCA cycle. Yellow, carbon flux from lactate to glucose via PC. **b** Hepatic glucose isotopologues, 15 min after [U-^13^C]-lactate injection. **c**–**e**, Relative abundance of hepatic glucose [M + 2] and glucose [M + 3] (**c**), the TCA cycle metabolites αKG [M + 2], succinate [M + 2] and malate [M + 2] (**d**), and total pyruvate and pyruvate [M + 3] (**e**) 15 min after [U-^13^C]-lactate injection. Metabolite peak area values were normalized to total ion count. **f**, **g** Hepatic lactate [M + 3] / pyruvate [M + 3] peak area ratio (**f**), and ratio of total liver metabolite levels of lactate/pyruvate (**g**) 15 min after [U-^13^C]-lactate injection. For (**b**–**g**), mice were at the ages of 5–7 months (young) and 20–24 months (old). *n* = 5 mice; bars represent mean ± SEM. One-way ANOVA with Fisher’s LSD method. Exact *p*-values are reported in the Source Data file. *p < 0.05, **p < 0.01, ***p < 0.001, ****p < 0.0001. **h** Pyruvate tolerance test in 20 months old 6 h-fasted WT (*n* = 7 mice) and SIRT6-tg (*n* = 6 mice) male mice.

With age, WT mice showed reduced GNG capacity even when supplemented with exogenous GNG precursors such as lactate and glycerol (Fig. 3c, d). Thus, the age-dependent GNG decline cannot be explained solely by limitations in substrate availability. To further understand the mechanism underlying the age-dependent reduction in GNG, in vivo stable isotope tracing using uniformly labeled ^13^C lactate was performed (Fig. 6a). We chose lactate, as its oxidation contributes carbons to both GNG and the TCA cycle^54,55^, two pathways that were affected by aging and SIRT6 (Figs. 2–5). [U-^13^C]-lactate was injected into 6 h-fasted young and old WT and SIRT6-tg mice, followed by analysis of liver metabolite isotopologue abundances 15 min after injection. Total lactate concentrations and fractional abundance of its M + 3 isotopologue (lactate labeled on all 3 carbons) were similar between groups in plasma and liver (Supplementary Fig. 7a, b). These findings indicate that lactate transport into the liver is not affected by aging or by SIRT6. Lactate oxidation resulted in extensive labeling of liver pyruvate, alanine, TCA cycle metabolites, and AAs that are derived from the TCA cycle (Glu, Gln, Asp, Asn) (Supplementary Fig. 7c). Importantly, liver glucose was also labeled, providing a direct evidence for lactate-derived hepatic GNG (Fig. 6b). In comparison to WT mice, liver glucose abundance was higher in SIRT6-tg following lactate administration in an age-independent manner (Fig. 6b). In young mice, no significant differences were found in the abundance of labeled pyruvate [M + 3], TCA cycle metabolites αKG, succinate and malate [M + 2], or glucose [M + 2, M + 3], between genotypes (Supplementary Fig. 7d). Interestingly, steady-state levels of labeled glucose [M + 2 to M + 6], and most notably, glucose M + 2 and M + 3 isotopologues significantly decreased in aged WT mice, while remaining higher in old SIRT6-tg (*p* = 0.06 and 0.11, respectively; Fig. 6c and Supplementary Fig. 7e). Labeling of the TCA cycle intermediates αKG, succinate, and malate significantly decreased with age in WT mice but remained higher in SIRT6-tg mice (Fig. 6d). Thus, direct incorporation of lactate’s carbons to the TCA cycle and to GNG deteriorates with age in WT mice but is rescued in old SIRT6-tg mice.

In liver, when there is excess lactate or upon GNG induction, lactate dehydrogenase (LDH) catalyzes the reversible conversion of NAD^+^-dependent lactate to pyruvate. With age, a significant reduction in total pyruvate and pyruvate M + 3 levels was found after [U-^13^C]-lactate injection (Fig. 6e). Interestingly, in comparison to old WT mice, total pyruvate and pyruvate M + 3 levels were higher in old SIRT6-tg mice (Fig. 6e and Supplementary Fig. 7c). These findings suggest that LDH production of pyruvate is higher in old SIRT6-tg mice, potentially due to higher NAD^+^ levels (Fig. 5f). The lactate/pyruvate ratio can be used as a proxy for the cytosolic NADH/NAD^+^ ratio^56^. Indeed, [U-^13^C]-lactate injection led to increased lactate[M + 3]/pyruvate[M + 3] and total lactate/pyruvate ratios with advanced age, while remaining significantly lower in old SIRT6-tg livers (Fig. 6f, g). These results suggest that SIRT6 preserves a lower cytosolic NADH/NAD^+^ ratio at old age, which drives the lactate-to-pyruvate direction of LDH reaction. To test this hypothesis, we injected pyruvate to old WT and SIRT6-tg mice, thus bypassing LDH reaction, and measured blood glucose. Interestingly, in contrast to lactate (Fig. 3c), no difference in GNG capacity from pyruvate was observed between genotypes (Fig. 6h). In addition, no differences in the ratios of malate[M + 2]/malate[M + 3] and aspartate[M + 2]/aspartate[M + 3] were found between age or genotypes (Supplementary Fig. 7f), suggesting that relative pyruvate carboxylase (PC) flux is not altered, but rather the upstream LDH-redox effect is prominent. Thus, maintenance of young-like redox balance by SIRT6 likely activates LDH towards lactate oxidation resulting in young-like lactate oxidation which enables maintenance of TCA cycle and gluconeogenic capacity at old age.

### SIRT6 promotes hepatic gluconeogenesis via increased lipolysis in adipose tissue

**Fig. 7:**
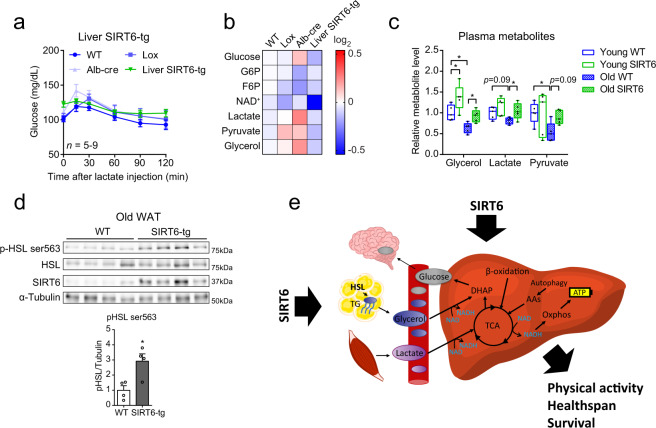
Enhanced adipose tissue-derived glycerol contributes to maintenance of normoglycemia in aged SIRT6-tg mice. **a** Lactate tolerance test in 22–24 months old liver-specific SIRT6-tg and appropriate littermate control male mice. Values are mean ± SEM of *n* = 6 WT mice, *n* = 5 lox mice, *n* = 9 Alb-cre mice and *n* = 5 liver SIRT6-tg mice. Two-way ANOVA showed no difference between liver SIRT6-tg and controls. Bars represent mean ± SEM. **b** Heatmap of GNG-related hepatic metabolites in 23–25 months old liver SIRT6-tg male mice and control littermates. Each square represents average metabolite abundance of *n* = 5 mice per genotype. See Supplementary Table 9 for source data. **c** Levels of the GNG precursors glycerol, lactate and pyruvate in the plasma of 6 h-fasted young (5–7 months) and old (20–24 months) mice, *n* = 5, males. Box extends from the 25th to 75th percentiles, line in the middle of the box is the median and whiskers go down to the smallest value and up to the largest. **d** Western blot and ImageJ quantification of HSL phosphorylation on Ser563 in white adipose tissue of 25 months old WT and SIRT6-tg littermates. *n* = 4 mice per genotype. Bars represent mean ± SEM, two-tailed Student’s *t*-test. For (**c**, **d**), exact *p*-values are reported in the Source Data file, *p < 0.05. **e** Schematic model depicting how SIRT6 improves longevity and healthspan by preserving NAD^+^ metabolism and energy production pathways in old age. Hepatic TCA cycle and GNG deteriorates during aging, leading to perturbed energy homeostasis and fasting glycemia in old age. SIRT6 overexpression increases hepatic energy production from fatty acids and AAs in the liver. This potentially spares GNG substrates, such as lactate and glycerol, whose shuttling during fasting from skeletal muscle and adipose tissue to the liver is increased by SIRT6. This cascade of events leads to preserved hepatic glucose production in old age. TG, triglyceride; HSL, hormone-sensitive lipase; DHAP, dihydroxyacetone phosphate; AAs, amino acids; Oxphos, oxidative phosphorylation.

GNG is regulated through both hepatic and extrahepatic mechanisms^13^. To determine whether SIRT6 also affects GNG and normoglycemia through extrahepatic mechanisms, liver-specific SIRT6-tg mice were generated and let to age (Supplementary Fig. 8a). In comparison to WT mice, whole-body SIRT6 overexpression mice have higher GNG capacity from lactate, increased hepatic GNG gene expression, and higher levels of key hepatic GNG-related metabolites (Figs. 3c, 4e, and 5c). However, liver-specific SIRT6-tg and control mice showed similar blood glucose levels after lactate injection, both at young and old ages (Fig. 7a and Supplementary Fig. 8b). In addition, liver-tg and control mice showed similar expression levels of key hepatic GNG genes, together with similar and even somewhat reduced levels of hepatic GNG-related metabolites (Fig. 7b, Supplementary Fig. 8c, and Supplementary Table 9). These findings show that increased SIRT6 activity in extrahepatic tissues is necessary to activate GNG, and emphasize the importance of SIRT6 in synergizing multiple tissues to promote GNG.

Differences in hormonal regulation or alterations in GNG precursor availability are two possible mechanisms underlying the extrahepatic effect of whole-body SIRT6 overexpression on GNG. No differences were found in insulin, glucagon, and corticosterone levels, the three known GNG regulators, between old WT and whole-body SIRT6-tg mice, at different fasting time points (Supplementary Fig. 8d). In addition, no difference in glucose-stimulated insulin secretion (GSIS) was observed between old WT and SIRT6-tg mice (Supplementary Fig. 8e). These results indicate that the effect of SIRT6 on GNG is not mediated by change in extrahepatic hormonal regulation.

A second extrahepatic mechanism that can activate GNG is substrate shuttling to the liver. The circulating levels of the GNG precursors lactate and pyruvate decreased with age in WT mice, but not in SIRT6-tg mice (Fig. 7c). Similarly, in WT mice, the levels of the liver GNG precursors lactate, pyruvate, and glycerol also decreased with age (Fig. 5c and Supplementary Fig. 6c). During fasting, glycerol and FFAs are released from adipose tissue as lipolysis products and glycerol is used for GNG in the liver. In addition, FFAs themselves can also stimulate hepatic GNG^57^. In comparison to fasted WT mice, 15 months old SIRT6-tg littermates have higher circulating glycerol levels (Fig. 2c). Thus, we hypothesized that in old age, SIRT6 enhances lipolysis and glycerol release from adipose tissue, contributing to increased substrate availability for GNG in the liver. Plasma glycerol levels in 6 h-fasted WT mice decreased with age. Yet, strikingly, old SIRT6-tg mice showed young-like plasma glycerol levels (Fig. 7c). Moreover, the active phosphorylated form of the key lipolytic enzyme, hormone-sensitive lipase (HSL), in the adipose tissue, increased in old SIRT6-tg mice (Fig. 7d). Old SIRT6-tg mice also showed higher levels of 16 h-fasted serum FFAs (Supplementary Fig. 8f). These findings show that enhanced lipolysis and glycerol release from adipose tissue likely contribute to normoglycemia in aged SIRT6-tg mice.

Altogether, SIRT6-tg mice maintain young-like energy homeostasis at old age. This occurs by increasing systemic GNG substrate availability and supporting their hepatic utilization by enhancing de novo synthesis of NAD^+^, a key modulator of healthy aging.

## Discussion

SIRT6 was previously shown to extend lifespan in male mice and improve health parameters in aged CB6 mixed-background animals^29,30^. Yet, essential questions about the broad effect of SIRT6 on healthy aging remained unanswered. Is the effect strain or sex dependent? How does SIRT6 promote healthy lifespan at the biochemical and molecular levels? And finally, how many tissues must overexpress SIRT6 to achieve these health improvements? To address these issues, we followed the effect of SIRT6 overexpression in inbred C57BL/6JOlaHsd mice. Here, we show that male and female C57BL/6JOlaHsd mice overexpressing SIRT6, but not SIRT1, live significantly longer than WT littermates. SIRT6-tg mice maintain young-like physical activity and metabolic flexibility, along with reduced aging-related pathologies. Moreover, further analyses showed that old SIRT6-tg mice show a young-like liver metabolite profile. Notably, SIRT6 enables energy production under limited energy conditions, such as fasting and aging. To mediate this function, SIRT6 promotes hepatic β-oxidation, lactate and glycerol shuttling and hepatic utilization, NAD^+^/NADH ratio in the liver, and glycerol release from adipose tissue. These SIRT6-regulated metabolic pathways coordinate to maintain young-like TCA cycle and GNG activities in old age (Fig. 7e). Thus, the positive effect of SIRT6 on healthy lifespan is strain and sex independent and requires SIRT6 regulation of energy production in at least two sites, liver and adipose tissues. Together, this emphasizes the potential of SIRT6-based therapeutic approaches in addressing age-related frailty and other diseases.

Previously, we showed that SIRT6 levels increase under fasting or CR^20^, suggesting a role for SIRT6 in regulating aging. Yet, animals in the wild usually die young, before aging occurs^58^. Hence, from an evolutionary perspective, natural selection likely does not directly influence aging. Therefore, SIRT6 most likely did not evolve as a regulator of aging, and the increase in its level upon fasting should be explained. Here, we show that during fasting, SIRT6 enables better production of energy, suggesting the evolutionary basis for the increase in SIRT6 levels upon limited availability of energy sources. Moreover, as seen in Fig. 3, glucose production capacity significantly decreases with age, an effect mitigated by increased SIRT6. Interestingly, Gorbunova and colleagues recently suggested a positive correlation between SIRT6-dependent DNA repair capacity and rodent lifespan^59^. Thus, it would be of great interest to examine the correlation between SIRT6-dependent energy production capacity and lifespan across evolution.

In addition to whole-body SIRT6 overexpression and deletion, several SIRT6 tissue-specific models were described. Adipose-specific SIRT6 deletion promotes insulin resistance^60^, HFD-induced obesity, inhibits lipolysis^61^, and impairs brown adipocyte thermogenic function^62^. In addition, increasing adipose SIRT6 levels using viral injection increases ATGL expression, suggesting higher lipolysis^61^. Liver-specific deletion of SIRT6 in mice results in significant metabolic alterations, such as increased triglyceride synthesis and fatty liver formation^34^. Whereas all these SIRT6-related phenotypes are consistent with our study, the reports regarding the effect of liver-specific SIRT6 deletion or overexpression on GNG are less clear. Dominy et al. showed that mice with shRNA ablation or adeno-viral overexpression of SIRT6 exhibit induction or inhibition of GNG, respectively^36^. In contrast, Deng and his colleagues reported that liver-specific SIRT6 deletion has no effect on GNG^34^, and our results show no difference in GNG from lactate at old age, and modest GNG inhibition at young age, in liver-specific SIRT6-tg mice (Fig. 7a and Supplementary Fig. 8b). Here, we show that whole-body SIRT6 overexpression promotes GNG from lactate and glycerol in aged mice. This emphasizes the requirement for additional tissue/s, besides the liver, in the process of SIRT6-mediated GNG induction. Indeed, in whole-body SIRT6 overexpression mice there is an increase in glycerol release from adipose tissue, which is used for GNG in the liver. This effect, together with our glycerol and lactate tolerance tests and lactate tracing results (Figs. 3 and 6) suggest a model where overexpression of SIRT6 both in the liver and adipose tissues is required for GNG induction.

Aging is often accompanied by a significant increase in frailty^63^. Individual frailty is based on five key areas: unintended weight loss, exhaustion, weakness, slow walking speed, and low physical activity^63^. Metabolic dysfunction, particularly in the ability to produce energy, is a major underlying mechanism of this syndrome. In addition to FFA β-oxidation, two primary SIRT6-dependent energy production pathways were identified here, the activation of TCA cycle and GNG. Interestingly, like SIRT6-tg mice, CR mice or rhesus monkeys also showed evidence for increased GNG^46,64,65^. Increased GNG along with these energy production pathways affects various aspects of healthy aging, primarily the capacity for physical activity^66,67^. In addition, a healthy metabolic state positively influences memory and sleep quality, which decline with age^68^.

The expression of the key hepatic GNG genes *Pck1* (encoding for PEPCK protein)*, Pcx*, *G6pc*, and *Fbp1* were higher in SIRT6-tg livers (Fig. 4e). Likewise, the levels of PEPCK significantly increased in CR mice^69^. This suggests that PEPCK is a key regulator of longevity and is potentially involved in preserving physical activity in old age. Indeed, a set of previous observations support this model. PEPCK knockdown or overexpression, reduces or increases *C. elegans* lifespan, respectively^70,71^. Moreover, PEPCK overexpression restores locomotor activity in old worms^71^. Thus, SIRT6 increases GNG capacity in parallel to normal glucose tolerance, promoting healthy aging of the liver and the organism. These findings suggest targeting GNG capacity with age as a therapeutic approach for the treatment of age-related frailty.

It is fascinating that SIRT6 also supports energy production through additional pathways. SIRT6 increases mitochondrial biogenesis (Fig. 4e), and NAD^+^ levels (Fig. 5f). In comparison to WT mice, mtDNA copy number was significantly higher in transgenic mice. The increased mitochondria number is potentially due to increased PGC1α levels (Fig. 4e). Yet, PGC1α might not be the sole contributor to increased mitochondria biogenesis, since increased PGC1α was found only in old transgenic mice. Yet, two major lines of evidence support increased PGC1a activity in young SIRT6-tg mice: first, we found a significant increase in mitochondrial copy number and second, PGC1α is a major activator of PPARα. Indeed, PPARα-dependent transcription, and β-oxidation, a PPARα-dependent pathway, were significantly higher in young SIRT6-tg mice. Thus, the potential activation of PGC1α activity in whole-body SIRT6 overexpression should be further explored.

Hepatic mRNA expression analysis showed that increased SIRT6 results in a repressed inflammatory signature, especially in males. These findings are in agreement with the well-documented anti-inflammatory role of SIRT6^31^. Interestingly, we found an increase in hepatic essential PUFA levels (Supplementary Fig. 6h), which are used by the liver as fuel and for anti-inflammatory signaling^72^. Thus, this suggests a mechanism underlying the anti-inflammatory function of SIRT6.

SIRT6 overexpression significantly enhances lifespan of both sexes in the inbred C57BL/6JOlaHsd mice. Yet, when overexpressed in mixed-CB6 background, SIRT6 led to lifespan extension only in males^29^. Two potential mechanisms can be suggested for this difference. First, while in CB6, SIRT6 overexpression lowered circulating IGF-1 levels only in males^29^, here serum IGF-1 was decreased by SIRT6 in both sexes (Supplementary Fig. 1i). Given the fundamental role of insulin/IGF-1 pathway in lifespan regulation^6^, this may be a prominent effect. Interestingly, SIRT6’s lifespan extension was stronger in males than in females (Fig. 1a). Accordingly, circulating IGF-1 levels are lower in WT females relative to WT males (Supplementary Fig. 1i). This masks the beneficial effect of SIRT6 on females’ lifespan via IGF-1. Second, weight maintenance at old age is associated with increased survival in mice and humans^38,39^. Interestingly, here, SIRT6 led to improved weight maintenance in females (Supplementary Fig. 1d, f). Thus, one can suggest that female-specific better weight maintenance contributes to SIRT6-dependent lifespan extension in females. Since in CB6 mice, this parameter was not followed longitudinally in our previous study, future research is required to examine weight maintenance in CB6 background as well.

SIRT6 had a milder effect in females in additional parameters, such as treadmill endurance running and liver transcriptional profile (Fig. 4a and Supplementary Fig. 2i, j). Endurance activity is an energy-demanding process. Thus, the improved endurance performance in males might be due to their stronger activation of the GNG and β-oxidation energy production pathways by SIRT6 (Figs. 3 and 4b). In comparison to young females, young male mice show a robust SIRT6-mediated hepatic transcriptional profile. This change is largely related to a higher basal inflammatory state in males, which is inhibited by SIRT6 (Fig. 4b). Interestingly, in humans, males are more susceptible to chronic inflammatory diseases than women prior to menopause^73^. Therefore, this might also contribute to the stronger SIRT6-dependent lifespan extension observed in males. Moreover, all these can also explain the stronger SIRT6 effect on lifespan in the current genetic background.

Liver NAD^+^ levels were significantly higher in old SIRT6-tg mice. This could be due to increased production or reduced utilization of NAD^+^. In addition, SIRT6-tg mice showed a significant increase in the levels of NAM, a reaction product of SIRT6. Increased NAM levels, suggest that NAD^+^ utilization by SIRT6 increases. Yet, as seen in Supplementary Fig. 6j, a reduced NAD^+^ usage by other consumers, due to reduced DNA damage or PARP1 activity was excluded. Increased NAM levels can support the increased NAD^+^ levels via two pathways. First, within the NAD^+^ salvage pathway, NAM can be used as a direct NAD^+^ precursor. Yet, no significant change in the levels of NAMPT, a key enzyme of NAD^+^ salvage, was found between WT and SIRT6-tg mice (not shown). Thus, increased NAM probably does not support the increased NAD^+^ levels via the salvage pathway. Second, recently, Mitchell et al. showed that increased NAM levels induces the NAD^+^ de novo synthesis pathway^53^. Indeed, the expression and protein levels of major enzymes in the de novo synthesis pathway were significantly higher in SIRT6-tg mice (Fig. 5g, h). Thus, we suggest that SIRT6 promotes de novo NAD^+^ synthesis, which can be used by various mediators of healthy aging such as other sirtuins and PARP’s under genotoxic stress.

Interestingly, the SIRT6-mediated increased hepatic NAD^+^ and cytosolic NAD^+^/NADH ratio favor the reverse LDH reaction. This serves as a central mechanism in GNG maintenance in old age by SIRT6, as revealed by hepatic lactate/pyruvate ratios and lactate and pyruvate tolerance tests (Figs. 3 and 6). The increased hepatic GNG gene expression in SIRT6-tg (Fig. 4e) is not the driving mechanism for GNG activation in this model.

Altogether, SIRT6 controls lifespan and the ability to generate energy at times of its limited availability, such as physical activity, fasting and aging. These pathways, together with SIRT6’s known regulating role of the key aging-related metabolic signaling pathways mTOR, IGF-1, and AMPK places SIRT6 as a master regulator of healthy aging and as a potential therapeutic target to preserve function and delay the onset of frailty.

## Methods

### Animal husbandry

All experiments were conducted in accordance with Bar-Ilan Institutional Animal Care and Use Committee and approved by the Ministry of Health of Israel. Mice were group housed in IVC cages (up to 5 animals/cage) in a specific pathogen-free environment and fed standard chow diet (Altromin 1324; total pathogen free, irradiated with 25 kGy) and water ad libitum. Animal rooms were maintained at 21–24 °C and 35–75% relative humidity, with 12/12 h (7 a.m. to 7 p.m.) dark–light cycle. Cages were routinely replaced every 10–14 days.

### Mice

Transgenic lines were originally generated in hybrid genetic backgrounds^26,74^, and were backcrossed for at least 10 generations to C57BL/6JOlaHsd background before usage. As several lines of SIRT6-tg mice were generated, it is important to note that all of the mice used in this study were from the same line (#55) as previously published^29^. SIRT1 + 6-tg mice were obtained by crossing SIRT6-tg with SIRT1-tg mice. Further matings were either of WT C57BL/6JOlaHsd (purchased from Envigo, Israel) with SIRT1 + 6-tg or of SIRT6-tg with SIRT1-tg. Mice overexpressing SIRT6 specifically in the liver were obtained by crossing Alb-cre mice (Jax Stock No: 003574) with lox-stop-lox SIRT6-knockin mice (Described in Figure S7A). To avoid potential epigenetic effects of the transgenes, progenies were obtained by alternate matings of male from one genotype with female from the other genotype and vice versa. At the age of 1 month, progenies were separated by sex, ear tagged, and distal tail (~2 mm) was cut for genotyping determination. WT and transgenic mice were housed in the same cage, and for all experiments, only littermates were compared. Throughout the study, if not stated otherwise, mice at the ages of 4–7 months were considered as young, and mice at the ages of 20–25 months were considered old. Mice with visibly unhealthy appearance were not used for in vivo experiments or for tissue collection.

### Lifespan

For lifespan study, mice were housed as described in “Animal husbandry” section and were left undisturbed in cages until natural death. These animals were not used for any other experiment and were virgins, the only procedure they underwent was the standard ear-tagging and 2 mm tail cutting for genotyping at 1 month of age. Other cohorts where raised for in vivo experiments and tissue collections. Mice were inspected daily for health issues, and any death was recorded. Animals showing significant signs of morbidity, based on the AAALAC guidelines, were euthanized for humane reasons, and were used for lifespan analysis since they were deemed to live to their full lifespan. No mice were censored from analysis. Lifespan was analyzed by Kaplan–Meier survival curves, and *p-*values were calculated by log-rank test using SPSS (version 20, IBM). Maximum lifespans and 20th percentiles were calculated as the proportion of each group still alive when the total population reached 90% or 20% mortality, respectively, and significance was analyzed with Fisher’s exact test.

### Histopathology

Histopathological analysis was performed on two separate mouse cohorts. The first cohort were mice sacrificed at age of 25 months by CO_2_ inhalation. From ~10 tissues, healthy-appearing part was taken into liquid nitrogen for protein/RNA extraction, and the remaining tissue parts together with the mouse’s backbone were transferred to 4% neutral formalin for histopathological analysis. The 2nd cohort was end-of-life examination (not from the cohort used for lifespan). Old animals found moribund, soon before estimated natural death, were used; in that case they were euthanized and tissues were fixed. Mice found after death could not be used for analysis due to poor quality of tissues.

Tissue samples were fixed in 4% neutral formalin, embedded in paraffin, sectioned 3 µm thick, and dried. Slides were de-waxed and re-hydrated through a series of graded ethanol and were stained with hematoxylin-eosin (H&E). Photographs were taken using an Olympus DP73 digital camera, and analyzed by an expert pathologist in a blinded fashion with respect to the genotypes of the analyzed samples.

Any of the following pathologies was considered as neoplasia: hyperplasia, lymphoma, sarcoma, hemangiosarcoma, hemangioma, adenoma, or carcinoma. Either lymphoma, sarcoma, hemangiosarcoma, or carcinoma were defined as cancer.

### Tissue and plasma collection

In all cases, mice were sacrificed between 2 p.m. and 4 p.m. to avoid any potential circadian effects on downstream analysis. For protein, RNA and DNA extraction, tissues were collected from mice fasted for 4 h (~10 a.m.–2 p.m.) and sacrificed by CO_2_ inhalation. Tissues were collected quickly (~5 min/mouse), and immediately snap-frozen in liquid nitrogen and stored at −80 °C prior to use. For liver metabolomics, mice fasted for 6 h (~8 a.m.–2 p.m.) or mice 15 min after [U-^13^C]-lactate injection (at ~3 p.m.) were sacrificed by cervical dislocation. Tissues were collected rapidly (~2 min/mouse for most tissues, liver was collected in ~30 s), snap-frozen in liquid nitrogen, and stored at −80 °C prior to use. For the analysis of plasma metabolites, ~30 µl of blood was drawn from tail bleeds into heparin-coated tubes immediately before mice’s sacrifice. Blood was centrifuged for 10 min at 2000*g* at 4 °C, plasma was transferred to new tube, snap-frozen in liquid nitrogen, and kept at −80 °C until use.

### Western blotting

Tissue homogenization was performed using the Bullet Blender homogenizer (Next Advance) in ice-cold lysis buffer (10 mM TrisHCl pH 7.4, 150 mM NaCl, 1 mM EDTA, 1 mM EGTA, 0.5% TritonX100, 7 M urea, 2 mM vanadate, 2.5 mM sodium pyrophosphate, 10 mM NaF, proteinase inhibitor cocktail pill [Santa Cruz Biotechnology]). Samples were centrifuged at 17,000*g* for 5 min at 4 °C to remove undissolved tissue debris, supernatant was collected, and centrifuged again at the same conditions to get rid of undissolved fat. The clear supernatant was collected and protein concentration was determined with Bradford protein assay (Bio-Rad). Samples were boiled with sample buffer for 7 min and equal amount of protein were run in polyacrylamide gels. Transfer was performed using Trans-Blot Turbo System and 0.2 µm nitrocellulose membrane (Biorad). Membranes were blocked for 1 h with 5% BSA and incubated overnight at 4 °C with primary antibody directed against β-actin (C4, sc-47778, at 1:1000 dilution), Ub (P4D1, sc-8017, 1:1000), PARP1 (F-2, sc-8007, 1:1000), α-tubulin (Sigma Aldrich clone B-5-1-2, T5168, 1:7000), SIRT6 (D8D12, cst-12486, 1:1000), PSMB5 (β5) (D1H6B, cst-12919, 1:1000), PSMB8 (β5i) (D1K7X, cst-13635, 1:1000), LC3B (cst-2775, 1:1000), p62 (cst-5114, 1:1000), HSL (D6W5S, cst-18381, 1:1000), phospho-HSL (ser 563) (cst-4139, 1:1000), phospho-Histone H2A.X (Ser139) (MilliporeSigma clone JBW301, #05-636, 1:200), PAR (Trevigen, 4335-AMC-050, 1:1000), NMNAT1 (ab45652, 1:1000), and TDO2 (LSBio, LS‑C748245, 1:500). Anti-mouse or anti-rabbit HRP-conjugated antibodies (Jackson ImmunoResearch, 115-035-146 and 111-035-003, 1:15000) were used as secondary antibodies. Bands were detected using Clarity™ Western ECL Substrate (Biorad) with pictures captured in Amersham Imager 680 (GE Healthcare). Quantification was performed by densitometric analysis using ImageJ software and normalization to α-tubulin, β-actin, or Ponceau S staining (Sigma-Aldrich).

### RNA extraction and quantitative real-time PCR

Liver tissues snap-frozen in liquid nitrogen upon dissection and further stored at −80 °C were cut to ~30 mg piece on dry ice, and homogenized with plastic pestles or in Precellys® Tissue Homogenizer in TRI Reagent (Sigma-Aldrich). RNA was extracted according to manufacturer’s specifications. RNA purity and concentration were assessed using a Thermo Fisher Scientific 2000 spectrophotometer (Thermo Fisher Scientific), and 1–3 µg were taken for cDNA synthesis (iScript™ Advanced cDNA Synthesis Kit, Biorad). The quantitative PCR was carried out using iTaq™ Universal SYBR® Green Supermix (Biorad) in a 15 µl total volume reaction containing 3 µl of 1:30 diluted cDNA. PCR amplification was performed using a StepOnePlus instrument (Applied Biosystems) or with CFX96 Real-Time-PCR detection system (Bio-Rad), and gene expression was normalized to *Actb* expression. The list of primers used for real-time PCR is given in Supplementary Table 10.

### Serum metabolomics

For serum metabolomics, we used male mice at age of 15 months. Blood was collected from facial vein of mice in the fed state, and then after 4 and 16 h fasting. Fed state was at Zeitgeber time 23, ~6 a.m., which is near the end of the mice’s normal feeding period. Mice were subsequently transferred to a new cage without food but with free access to water for up to 16 h. Blood was allowed to clot for 30 min at room temperature and centrifuged at 2000 *g* for 10 min at 4 °C to separate the serum. Collected serum was snap-frozen in liquid nitrogen and stored at −80 °C until later use. Metabolomics analysis was performed by gas chromatography time-of-flight mass spectrometry (GC-TOF-MS) in the UC Davis West Coast Metabolomics Center with the following procedure. First, 20 µl serum was mixed with 1 ml of extraction mix containing acetonitrile, isopropanol, and water in proportions 3:3:2 (JT Baker, Center Valley PA), then vortexed for 10 s and then 6 min at 4 °C. Samples were next centrifuged for 2 min at 14,000 *g*, 500 µl extract was transferred to a new tube and dried via evaporation overnight using speed vacuum concentration system (Labconco, Kansas City MO). The dried tube was resuspended in 500 µl of 50% acetonitrile, vortexed for 10 s, and centrifuged for 2 min at 14,000 *g*. Then, 475 µl extract was moved to a new tube and dried via evaporation as before. Samples were then derivatized by methoxyamine hydrochloride in pyridine and subsequently by *N*-methyl-*N*-trimethylsilyltrifluoroacetamide. Data acquisition was performed as previously published^45,75^. Briefly, the column used was Restek Corporation Rtx-5Sil MS (30 m length × 0.25 mm ID with 0.25 μm film made of 95% dimethyl/5% diphenylpolysiloxane) protected by a 10 m long empty guard column. MS parameters are used as follows: a Leco Pegasus IV mass spectrometer is used with unit mass resolution at 17 spectra/s from 80 to 500 Da at −70 eV ionization energy and 1800 V detector voltage with a 230 °C transfer line and a 250 °C ion source. Injection volume was 0.5 μl with10 μl/s injection speed and helium mobile phase. ChromaTOF version 2.32 software was used for data preprocessing without smoothing, 3 s peak width, baseline subtraction just above the noise level, and automatic mass spectral deconvolution and peak detection at signal/noise levels of 5:1 throughout the chromatogram. Apex masses are reported for use in the BinBase algorithm. Quantification is reported as peak height using the unique ion as default. Raw data were normalized to the sum of all peak heights for all identified metabolites, but not the unknowns, for each sample, and differences between groups where further analyzed using GraphPad prism software. Raw metabolomics MS data were deposited to NIH Common Fund’s National Metabolomics Data Repository (NMDR) website, the Metabolomics Workbench, https://www.metabolomicsworkbench.org where it has been assigned Project ID PR001005. The data can be accessed directly via its Project 10.21228/M8SH6G.

### Blood parameters

For blood hematology parameters, whole blood was taken from tail bleedings into EDTA-coated tubes and sent on ice for analysis at AML laboratories, Israel. Complete blood count was performed by Sysmex XS-1000i hematology analyzer, and differential count of white blood cells was performed manually by a veterinary in a blinded manner with respect to genotypes. For serum chemistry, blood was derived from facial vein of 3 h-fasted mice, except for total cholesterol and HDL that in old mice were analyzed in blood derived from heart puncture of 4 h-fasted mice. Blood was allowed to clot for 30 min at room temperature, centrifuged at 2000 *g* for 10 min at 4 °C to separate the serum and sent to AML laboratories, Israel, where serum biochemistry parameters were analyzed using Cobas 6000 automated analyzer (Roche Diagnostics). LDL levels were calculated by subtracting HDL levels from total cholesterol levels. Serum IGF-1 levels were determined using IGF-1 ELISA Kit (R&D Systems) following the manufacturer’s instructions.

### Transcriptomics

RNA was extracted using TRI Reagent (Sigma-Aldrich) and extraction quality was evaluated using Tapestation RNA Assay (Agilent Technologies, CA, USA). Libraries were prepared with the NEBNext Ultra RNA library prep Kit (#E7530, NEB) using manufacturer’s instructions, and final evaluation of library integrity was carried out with Tapestation DNA HS Assay (Agilent Technologies, CA, USA). Equimolar pooling of libraries were performed based on Qubit quantification, and loaded onto an Illumina Hiseq 2500 platform (Illumina, CA, USA). Libraries were sequenced in a multiplexed fashion using single-end sequencing protocol, yielding 24–47 million reads per sample. Reads were aligned to mouse genome (mm10) using the STAR RNA-seq aligner software (version STAR_2.5.0a)^76^. The STAR genome database was built with the Gencode annotation file (vM12). Only uniquely mapped reads (20–36 million reads per sample) were considered for further analysis. Raw read counts for 49585 Gencode-annotated gene-level features were determined using HTSeq-count^77^. Differentially expressed genes were determined with the R Bioconductor package DESeq2^78^, and *p*-values were corrected with Benjamini-Hochberg false discovery rate (FDR) procedure. For the analysis of predicted upstream regulators (Fig. 4d), we used Ingenuity Pathway Analysis software^79^ using input gene lists with fold change cutoff ±1.5 with FDR *q*-value < 0.1 for males and FDR *q-*value < 0.25 for females, yielding 2880 and 184 differentially expressed genes in males and females, respectively.

For pathway analysis, we used Gene Set Enrichment Analysis (GSEA)^80^. We uploaded expression dataset gene list containing 21,575 genes which were defined as expressed genes, these genes had minimal normalized mean read count of 0.5 reads/gene. Analysis was performed using the stringent option of 1000 permutations.

### Metabolic cages and treadmill running

In vivo metabolic performance and physical activity were measured with automated indirect calorimetry system (TSE Systems GmbH) using animals at the indicated age and sex. Mice were individually housed in home cages and were allowed to acclimate for 24 h. Then, food and water consumption, oxygen consumption (VO_2_), carbon dioxide production (VCO_2_), respiratory exchange ratio (RER), and spontaneous home cage activity were continuously recorded for 72 h. Spontaneous home cage activity is monitored using an infrared light-beam frame which records beam interruptions in the *x*–*y* axis. After the 72 h period, a running wheel was added to each cage and running distance, running time, average and max speed on wheel, were recorded for another 72 h period, of which the first 24 h were considered as acclimation period and not included in analysis. For treadmill running assay, mice were first acclimated to the treadmill for 3 constitutive days for 5 min at a speed of 5 m/min. At the 4th day, an incremental protocol was used: treadmill speed began at 5 m/min and was increased to 8 m/min after 2 min. Thereafter, the speed was increased at a rate of 2 m/min every 2 min and the distance (m) and time (min) to exhaustion, as defined by unwillingness to move along the treadmill for at least 5 s albeit stimulation of mild electric shock, were determined. The formula to determine the amount of work (*J*) performed was: *J* = mass (kg) × g (9.8 m/s^2^) × distance (m) × sin(θ) (with an incline of θ = 5°).

### Measurements of blood glucose during fasting

Blood glucose was measured by tail bleeds using glucometer test strips (Abbot). Few microliters of blood were drawn from the same individual mice at fed state, and at 4, 8, 12, 16, and 24 h of fast. Mice had free access to water along the experiment. Fed state was at Zeitgeber time 23, ~6 a.m.

### Intraperitoneal lactate, glycerol pyruvate, and glucose tolerance tests

Sodium L-lactate (Spectrum Chemicals), glycerol and sodium pyruvate (Sigma-Aldrich) were prepared as 0.1 mg/µl in PBS, glucose was prepared as 20% w/v in PBS, and were filtered with 0.2 µm filter. Mice at the indicated age and sex were fasted for 6 h (8 a.m.–2 p.m.) for lactate, glycerol, and pyruvate tolerance tests, or 16 h overnight for glucose tolerance test, weighted, and ~1 mm of tail end were cut with sterile scalpel. Blood glucose levels were measured by tail bleeding before or at the indicated time points after lactate, glycerol, pyruvate (all 1 g/kg body weight), or glucose (2 g/kg body weight) intraperitoneal injection using glucometer test strips (Abbot) by observer blinded to animal genotype. Animals had free access to water during the whole experiment.

### Glucose-stimulated insulin secretion (GSIS)

Mice were injected intraperitoneally with 2 g/kg glucose, and ~20 µl of blood were withdrawn from tail bleeding before and at the indicated time points after injection. Serum was separated and kept at −80 °C until analysis. Insulin levels in serum samples were measured with ultra-sensitive mouse insulin ELISA kit (Crystal Chem) following the manufacturer’s instructions.

### Body composition measurements

Body composition was measured by nuclear magnetic resonance (NMR) using the Minispec LF90 (Bruker Optics, Billerica, MA). Lean and fat mass were recorded.

### Hepatic glycogen measurement

Liver glycogen was measured using Glycogen Assay Kit (Sigma-Aldrich, MAK016) following manufacturer’s protocol.

### Serum FFA measurement

Serum FFAs were measured using Free Fatty Acid Quantification Kit (BioVision, K612) following manufacturer’s protocol.

### Liver and plasma metabolite measurements

To maximize accuracy and to take into account tissue inhomogeneity, metabolites were extracted from three separate liver pieces for each mouse, and the average metabolite value of the three technical replicates was calculated at analysis. In detail, frozen liver pieces weighting ~30 mg were transferred into soft tissue homogenizing CK 14 tubes containing 1.4 mm ceramic beads (Bertin corp.) prefilled with 1 ml of cold (−20 °C) metabolite extraction solvent (Methanol:Acetonitrile:H2O::50:30:20) and kept on ice. Samples were homogenized using Precellys 24 tissue homogenizer (3 × 20 s at 6000 rpm, with a 30 s gap between each of the three cycles, Bertin Technologies) cooled to 2 °C. Homogenized extracts were centrifuged in the Precellys tubes at 18,000 *g* for 15 min at 4 °C, supernatants were collected in microcentrifuge tubes and centrifuged again at 18,000 *g* for 10 min at 4 °C. The supernatants were transferred to glass HPLC vials and kept at −75 °C prior to LC-MS analysis. For the analysis of plasma glycerol and lactate, plasma was thawed on ice and mixed in 2 °C cooled Precellys 24 tissue homogenizer (3 × 30 s at 4500 rpm). Plasma was diluted in a ratio of 1:100 for lactate and pyruvate, or 1:10 for glycerol with cold (−20 °C) metabolite extraction solvent (Methanol:Acetonitrile::75:25) and mixed again in Precellys homogenizer (same conditions). Samples were then treated with similar centrifugations as liver homogenized extracts and kept at −75 °C prior to LC-MS analysis. Similar to liver, for each plasma sample metabolites were extracted in triplicate, with the exception of 3 samples that had too small plasma volume, 2 of them analyzed with 2 replicates and 1 sample that did not have replicates.

LC-MS metabolomics analysis was performed as described previously^81^. Briefly, Thermo Ultimate 3000 high-performance liquid chromatography (HPLC) system coupled to Q-Exactive Orbitrap Mass Spectrometer (Thermo Fisher Scientific) was used with a resolution of 35,000 at 200 mass/charge ratio (*m*/*z*), electrospray ionization, and polarity switching mode to enable both positive and negative ions across a mass range of 67–1000 *m*/*z*. HPLC setup consisted ZIC-pHILIC column (SeQuant; 150 mm × 2.1 mm, 5 μm; Merck), with a ZIC-pHILIC guard column (SeQuant; 20 mm × 2.1 mm). Here, 5 µl of Biological extracts were injected and the compounds were separated with mobile phase gradient of 15 min, starting at 20% aqueous (20 mM ammonium carbonate adjusted to pH 9.2 with 0.1% of 25% ammonium hydroxide) and 80% organic (acetonitrile) and terminated with 20% acetonitrile. Flow rate and column temperature were maintained at 0.2 ml/min and 45 °C, respectively, for a total run time of 27 min. All metabolites were detected using mass accuracy below 5 ppm. Thermo Xcalibur 4.0 was used for data acquisition.

TraceFinder 4.1 was used for analysis. Peak areas of metabolites were determined by using the exact mass of the singly charged ions. The retention time of metabolites was predetermined on the pHILIC column by analyzing an in-house MS metabolite library that was built by running commercially available standards. For data normalization of liver tissues, raw data files were processed with Compound Discoverer 3.0 to obtain total measurable ions peak area for each sample. Metabolite peak area values in each sample were divided by the total ion peak area value measured in the sample. Raw liver metabolomics MS data were deposited to NIH Common Fund’s National Metabolomics Data Repository (NMDR) website, the Metabolomics Workbench, https://www.metabolomicsworkbench.org where it has been assigned Project ID PR001017. The data can be accessed directly via its Project 10.21228/M87M4S.

### Quantification of mitochondrial DNA

Total DNA was isolated by standard proteinase K and phenol-chloroform methods. The copy number of mtDNA was analyzed by quantitative real-time PCR. Of the total DNA, 0.4 ng was used as a template for the amplification of mtDNA. Product levels from primers against mouse mtDNA (5′-AAGACACCTTGCCTAGCCACAC-3′ and 5′-TGGCTGGCACGAAATTTACC-3′) were normalized against nuclear 18S rRNA gene (5′-AACTTTCGATGGTAGTCGCCG-3′ and 5′-CCTTGGATGTGGTAGCCGTTT-3′).

### Liver proteomics

#### Sample preparation for LC-MS analysis

In order to remove lipids and detergents 300 μg of mouse tissue lysates from 6 controls and 6 cases were precipitated using standard methanol/chloroform extraction protocol (sample:methanol:chloroform:water::1:4:1:3)^82^. Proteins were resuspended in 30 μl of concentrated urea buffer (8 M urea, 2 M thiourea, 150 mM NaCl (Sigma)), reduced with 50 mM DTT for 1 h at 36 °C and alkylated with 100 mM iodoacetamide for 1 h at 36 °C in the dark. The concentrated urea/protein mixture was diluted 12 times with 50 mM ammonium bicarbonate buffer and proteins were digested for 18 h at 36 °C using trypsin/LysC mixture (Promega) in 1:50 (w/w) enzyme-to-protein ratio. Protein digests were desalted on 10 × 4.0 mm C18 cartridge (Restek, cat# 917450210) using Agilent 1260 Bio-inert HPLC system with the fraction collector. Purified peptides were speed vacuum dried and stored at −80 °C until further processing. Peptides (100 μg) were labeled with Tandem Mass Tags (TMT 6plex, Thermo Fisher) according to manufactures instructions. Each TMT labeling reaction contains 6 labels to be multiplexed in a single MS run. Labeled peptides from 6 different TMT tags were combined into one experiment and fractionated.

#### High-pH RPLC fractionation and concatenation strategy

High-pH RPLC fractionation was performed on Agilent 1260 Bio-inert HPLC system using 3.9 mm × 5 mm XBridge BEH Shield RP18 XP VanGuard cartridge and 4.6 mm × 250 mm XBridge Peptide BEH C18 column (Waters). Solvent composition was as follows: 10 mM ammonium formate (pH 10) as mobile phase A and 10 mM ammonium formate and 90% ACN (pH 10) as mobile phase B^83^. TMT-labeled peptides prepared from mouse livers were separated using a linear organic gradient (from 5% to 50% B in 80 min). Initially, 80 fractions were collected during each LC run at 1 min intervals each. Three individual high-pH fractions were merged into 15 master fractions with the 15 min intervals between each fraction (fraction 1, 16, 31, 46, 61 = master fraction 1, fraction 2, 17, 32, 47, 62 = master fraction 2, and so on). Combined fractions were speed vacuum dried, desalted and stored at −80 °C until final LC-MS/MS analysis.

#### Nano LC-MS/MS analyses

Purified peptide fractions were analyzed using UltiMate 3000 Nano LC Systems coupled to the Q Exactive HF mass spectrometer (Thermo Scientific, San Jose, CA). Each fraction was separated on a 35 cm capillary column (3 µm C18 silica, Hamilton, HxSil cat# 79139) with 150 μm ID on a linear organic gradient using 550 nl/min flow rate. Gradient went from 5% to 35% B in 90 min. Mobile phases A and B consisted of 0.1% formic acid in water and 0.1% formic acid in acetonitrile, respectively. Tandem mass spectra were obtained using Q Exactive HF mass spectrometer with the heated capillary temperature +280 °C and spray voltage set to 2.5 kV. Full MS1 spectra were acquired from 300 to 1500 *m*/*z* at 120,000 resolution and 50 ms maximum accumulation time with automatic gain control (AGC) set to 3 × 10^6^. Dd-MS2 spectra were acquired using dynamic *m*/*z* range with fixed first mass of 100 *m*/*z*. MS/MS spectra were resolved to 30,000 with 155 ms of maximum accumulation time and AGC target set to 2 × 10^5^. Twelve most abundant ions were selected for fragmentation using 30% normalized high collision energy. A dynamic exclusion time of 40 s was used to discriminate against the previously analyzed ions.

#### Bioinformatics analysis

Raw files from LC-MS analysis were converted to mascot generic format (.mgf) using MSConvert software (ProteoWizard 3.0.6002) and the spectra were searched with Mascot 2.4.1 and X!Tandem CYCLONE (2010.12.01.1) against SwissProt mouse sequences from the Uniprot database (version year 2016, appended with 115 known contaminant proteins). The search parameters were set as TMT6plex lysine and N-terminus as fixed modifications and variable modifications of carbamidomethyl cysteine, deamidation of asparagine and glutamate, carbamylation of lysine and N-terminus, and oxidized methionine. A peptide mass tolerance of 20 ppm and 0.08 Da, respectively, and two missed cleavages were allowed for precursor and fragment ions in agreement with the instrument mass accuracy. Mascot and X!Tandem search engine results were analyzed in Scaffold Q + 4.4.6 (Proteome Software, Inc). Peptide and protein probability were calculated by PeptideProphet and ProteinProphet probability model^84,85^. The TMT channels’ isotopic purity was also corrected according to the TMT Kit instructions.

Protein results were filtered at 1% thresholds of both protein and peptide FDR and required at least one unique peptide for protein identification. Those proteins that were identified from a single peptide were included in further analysis if that identification was confirmed by more than one search engine (as previously described) and identified across all samples. Reporter ion intensity quantitative values were extracted from Scaffold, and decoy spectra, contaminant spectra, peptide spectra shared between more than one protein and peptides with missing channel intensities were removed (overall < 2% missing quantification signals). The log2-transformed relative abundance was then normalized by median subtraction from all reporter ion intensity belonging to a protein across all channels. Relative protein abundance was estimated by the median of all peptides for a protein combined. Protein sample loading effects from sample preparations were corrected by median polishing, i.e., subtracting the channel median from the relative abundance estimate across all channels to have a median zero as described elsewhere^86,87^. Quantified proteins were clustered together if they shared common peptides and corresponding gene names were assigned to each protein for simplified data representation. Each protein from two TMT experiments were fitted to a linear model and empirical Bayes methods for assessing differential expression^87^ between groups. Protein significance (*p*-values) were calculated by ordinary *t*-test and moderate *t*-test across sample groups. The moderated *t*-statistic is the ratio of the log2 expression level of the protein to its standard error. Log fold expression change over sample groups were calculated and *p*-value < 0.05 for a protein was considered as significant. Annotation of the proteins were performed by manual curation and combining information from Uniprot, GO, and KEGG database. Further bioinformatics analysis and visualization of the data were performed using R programming language (3.4.0) and the free libraries available on Bioconductor.

### Statistical analysis

Data are presented as mean ± SEM for bar and line graphs. For box plots, box extends from the 25th to 75th percentiles, line in the middle of the box is the median and whiskers go down to the smallest value and up to the largest. *n* indicates the number of animals per experimental group, and is reported in the figures or figure legends. Statistical significance for single comparisons was calculated with unpaired two-tailed Student’s *t*-test. One-way or two-way analysis of variance (ANOVA) with fisher’s LSD method was used for multiple-group comparisons limited to up to 3 planned pairwise comparisons^88^. For multiple-group comparisons containing more than 3 pairwise comparisons, two-way ANOVA with Dunnett’s or Sidak’s post hoc test was used. For comparisons containing 3 variables (e.g., age, genotype, time), three-way repeated measures ANOVA with Sidak’s post hoc test was performed. Histopathology statistics values were derived with Fisher’s exact test. Pearson correlation coefficients were used to measure correlations. *t*-test values were calculated in Microsoft excel, one-way and two-way ANOVA were calculated with GraphPad prism software version 6.0, and three-way ANOVA was performed using SPSS (version 20, IBM). Heatmap of significant metabolites and Metabolite Set Enrichment Analysis (MSEA) was generated using the web-based MetaboAnalyst 4.0 software^89^ (https://www.metaboanalyst.ca/). All comparisons were two-tailed, and *p* < 0.05 was considered to be a statistically significant difference. *p < 0.05, **p < 0.01, ***p < 0.001, ****p < 0.0001.

### Reporting summary

Further information on research design is available in the Nature Research Reporting Summary linked to this article.

## Supplementary information

Supplementary Information

Reporting summary

## Data Availability

Raw and processed RNA-seq datasets were deposited to NCBI’s GEO database under the accession number: GSE157838, the data can be accessed using the link. The MS proteomics data have been deposited to the ProteomeXchange Consortium via the PRIDE^90^ partner repository with the dataset identifier PXD021447, and can be accessed using the link. All raw metabolomics MS data were deposited to NIH Common Fund’s National Metabolomics Data Repository (NMDR) website, the Metabolomics Workbench, https://www.metabolomicsworkbench.org where it has been assigned Project IDs PR001005 (serum metabolomics) and PR001017 (liver metabolomics). The data can be accessed directly via its Project DOIs 10.21228/M8SH6G and 10.21228/M87M4S. Databases used in this study: Uniprot (https://www.uniprot.org/), GO (http://geneontology.org/) and KEGG database (https://www.genome.jp/kegg/). Source data are provided with this paper.

## Source data

Source Data
