# Supplementary material for: Restoration of energy homeostasis by SIRT6 extends healthy lifespan
Source: Nat Commun. 2021 May 28;12:3208. doi: 10.1038/s41467-021-23545-7 (PMC8163764; doi:10.1038/s41467-021-23545-7)
Supplement: Supplementary file 1 — Supplementary Information [file 41467_2021_23545_MOESM1_ESM.pdf]

Supplementary Information for

Restoration of energy homeostasis by SIRT6 extends healthy lifespan

Roichman and Elhanati et al.

Supplementary Fig. 1

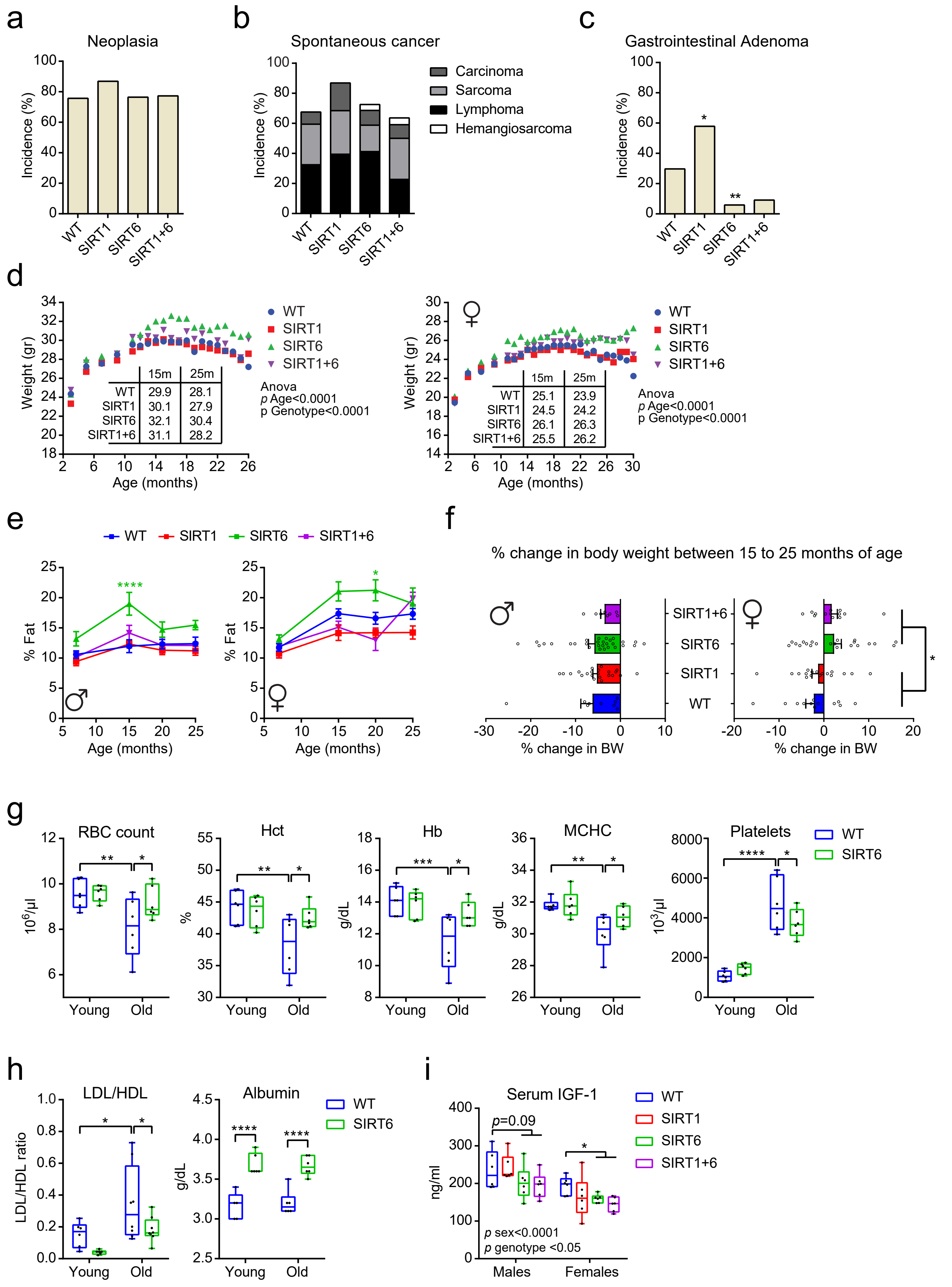

Supplementary Fig. 1. Extended healthspan parameters. (Related to Fig. 1)

a-c, Incidence of neoplasia (**a**), cancer (**b**) and gastrointestinal adenoma (**c**) at time of natural death. For **a-c**, *n*=37 WT, *n*=38 SIRT1-tg, *n*=51 SIRT6-tg, and *n*=22 SIRT1+6-tg mice, both sexes. Two-tailed Fisher's exact test. *, p<0.05 vs WT. Exact p values are reported in Supplementary Table 1. **d**, Median weight trajectories for males and females from the different genotypes. Inset: median weights at 15 months and 25 months of age. See Dunnett's multiple comparisons tests statistics in the Source Data file. **e**, Total body fat as a percentage of body weight, determined by NMR. Values are mean ± SEM for 9 mice per genotype (exceptions – *n*=8 for all groups at 25 months, for male WT and SIRT1+6 at 20 months, and for female SIRT1+6 at all ages, *n*=10 for male SIRT1 at 20 months and for male SIRT6 at 7,15 and 20 months), calculated by two-way Anova, Dunnett's post-hoc. *, p<0.05 vs WT, ****, p<0.0001 vs WT**. f**, Percentage body weight change between 15 and 25 months of age. For males *n*=9 WT, *n*=22 SIRT1, *n*=29 SIRT6, *n*=8 SIRT1+6, for females *n*=12 WT, *n*=17 SIRT1, *n*=21 SIRT6, *n*=13 SIRT1+6. Values are mean ± SEM, two-tailed student's t test, *, p<0.05. **g**, Red blood cell (RBC) count, hematocrit (Hct), hemoglobin (Hb), mean corpuscular hemoglobin concentration (MCHC) and platelet count in males; *n*=6 mice per group. **h**, Serum LDL/HDL ratio (*n*=6 young and *n*=8 old mice per genotype) and serum albumin (*n*=6 mice for all groups except young WT where *n*=5) in males. For **g** and **h**, ages are 5-7 months (young) and 24 months (old), two-way ANOVA with Fisher's LSD method. **i**, Serum IGF-1 levels measured in 16h overnight fasted animals; *n*=6, 6 months of age, and analyzed by two-way ANOVA (Sex, Genotype as independent variables) with Dunnett's post-hoc test. Exact p values are reported in the Source Data file. *, p<0.05, **, p<0.01, ***, p<0.001, ****, p<0.0001. For **g-i**, box extends from the 25th to 75th percentiles, line in the middle of the box is the median and whiskers go down to the smallest value and up to the largest.

Supplementary Fig. 2

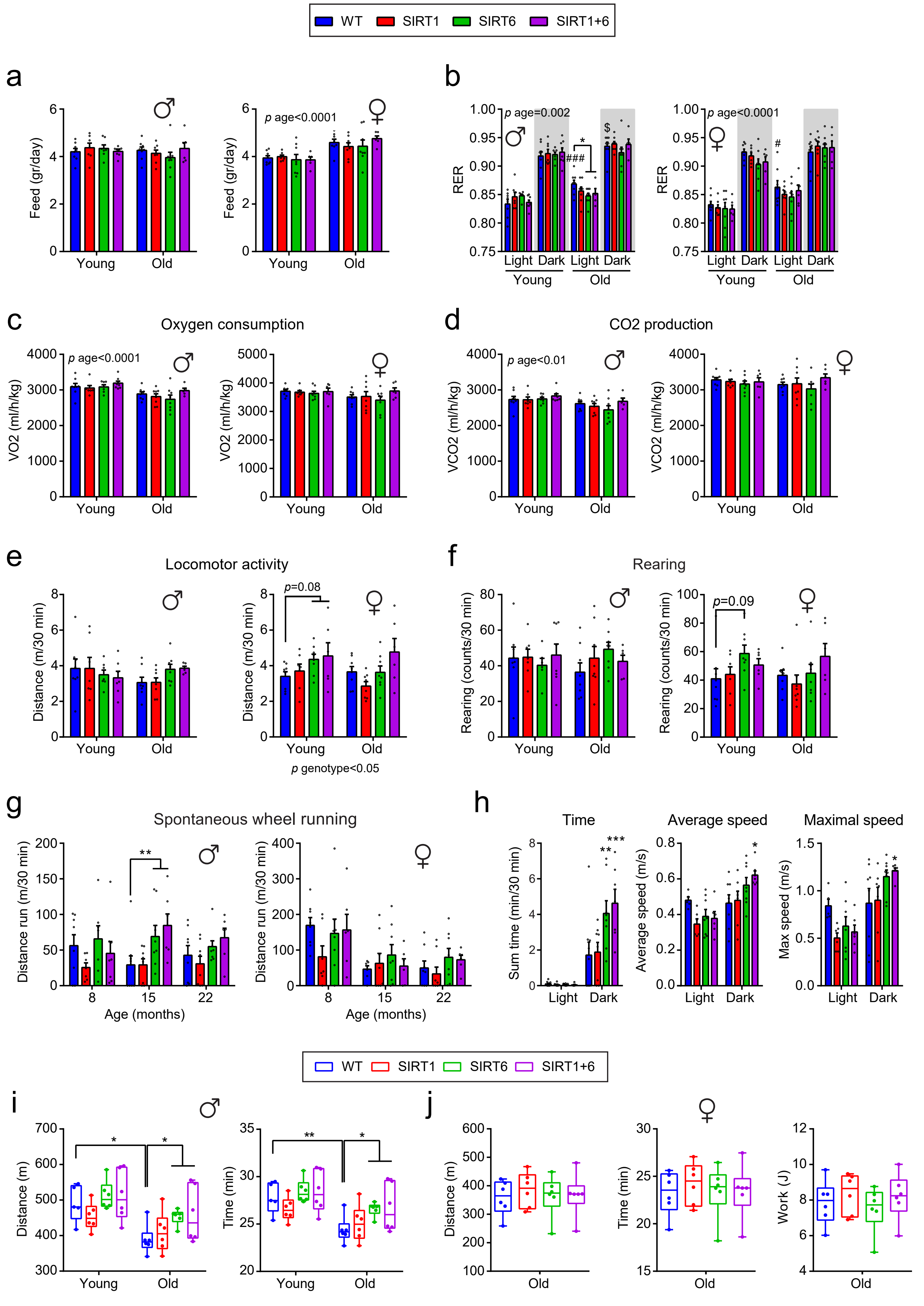

Supplementary Fig. 2. Extended metabolic cage and treadmill data. (Related to Fig. 1)

a, Food intake in young and old, male and female mice. **b**, Average RER in young and old males and females. Three-way ANOVA with Sidak's post-hoc test. #, $p<0.05$; ###, $p<0.001$ vs. young WT in light phase; $, $p=0.09$ vs. young WT in dark phase. **c-f**, Oxygen consumption (**c**), CO_2 production (**d**), spontaneous locomotor (**e**) and rearing (**f**) activities in young and old males and females. For **a-f**, ages are 8 (young) and 22 (old) months, *n*=8 WT mice, *n*=7 young SIRT1 mice, *n*=8 old SIRT1 mice, *n*=7 young SIRT6 male mice, *n*=8 female SIRT6 and old male SIRT6 mice, *n*=8 young male SIRT1+6 mice, *n*=6 old male SIRT1+6 mice in **a**,**e**,**f**, *n*=5 old male SIRT1+6 mice in **b**-**d**, *n*=6 female SIRT1+6 mice. Oxygen consumption and CO_2 production were normalized to total body weight. **g**, Distance of spontaneous wheel running at the indicated ages; *n*=8 mice per group, except of male WT at 15 months (*n*=9), female WT at 15 months, SIRT1 at 8 months, male SIRT6 at 8 months, male SIRT1+6 at 15 months (*n*=7), and SIRT1+6 females and SIRT1+6 males at 22 months (*n*=6). **h**, Total time, average speed and maximal speed during spontaneous wheel running at 15 months of age; *n*=9 WT mice, *n*=8 SIRT1 mice, *n*=8 SIRT6 mice, *n*=7 SIRT1+6 mice, males. For **a** and **c-h**, statistical significance was calculated using two-way ANOVA (age, genotype) with Dunnett's post-hoc test. **i**, Distance and duration of running on a treadmill by 6 months and 21 months old males; *n*=6 mice per group, except for young WT where *n*=5 mice, two-way ANOVA with Sidak's post hoc. **j**, Distance, time and work performed on treadmill by old females; one-way ANOVA with Fisher's LSD method, *n*=6 mice, 23 months. In **a-h** values are mean ± SEM. For **i**, **j**, box extends from the 25th to 75th percentiles, line in the middle of the box is the median and whiskers go down to the smallest value and up to the largest. Exact p values are reported in the Source Data file. For all panels, *, $p<0.05$, **, $p<0.01$, ***, $p<0.001$.

Supplementary Fig. 3

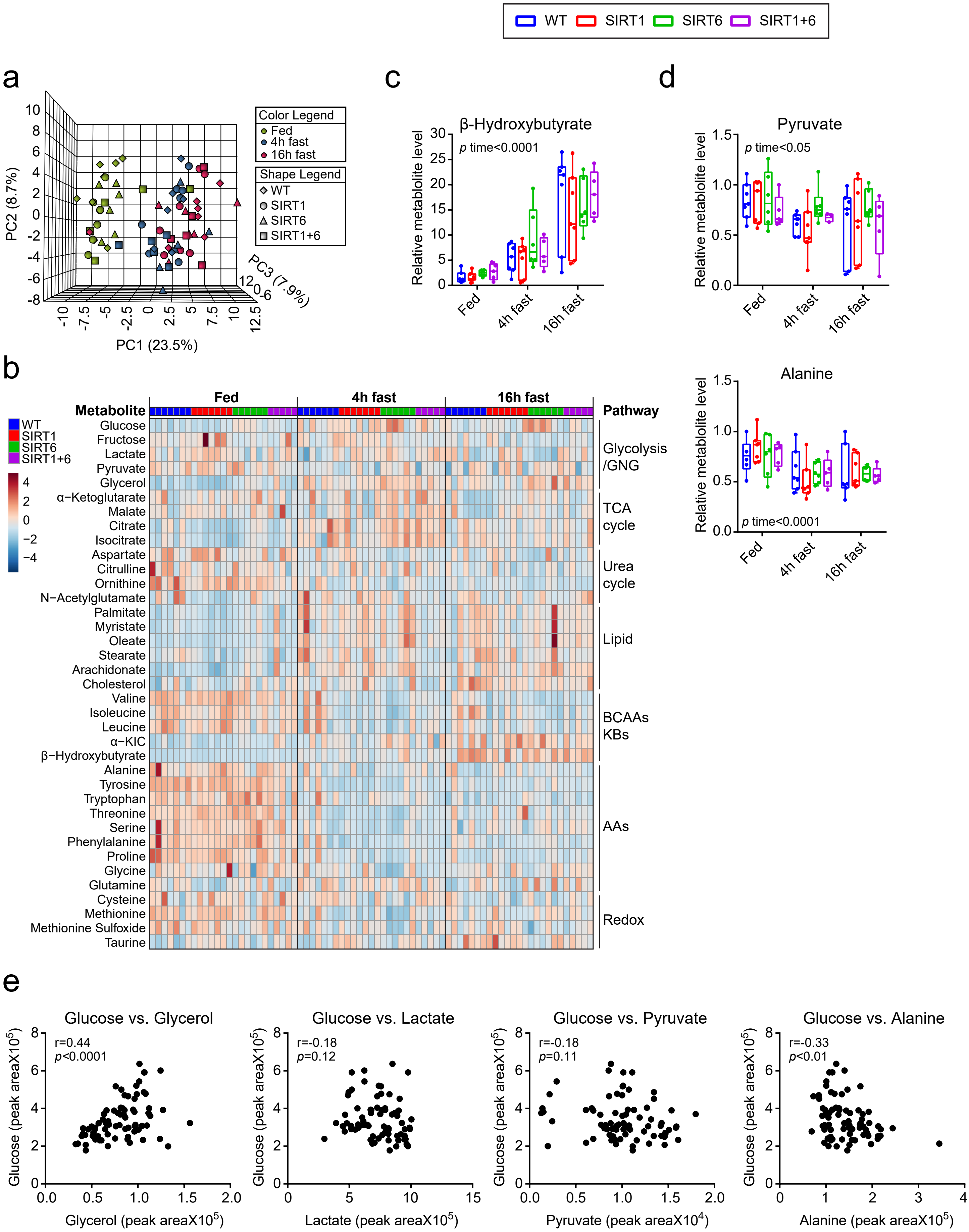

Supplementary Fig. 3. Serum metabolomics. (Related to Fig. 2)

a, PCA using the list of 130 identified metabolites in serum of WT and TG mice. **b**, Heatmap showing metabolites from key metabolic pathways that were significantly changed either by age, genotype or both, as calculated by two-way ANOVA. Each symbol represents an individual mouse. **c-d**, Scatterplots showing levels of the ketosis marker β-hydroxybutyrate (**c**), and the GNG precursors, pyruvate and alanine (**d**). n = 7 WT mice, n = 7 SIRT1-tg mice, n = 6 SIRT6-tg mice and n = 5 SIRT1+6-tg mice, two-way ANOVA (age, genotype) with Dunnett's pot-hoc test. Exact p values are reported in Supplementary Table 4. Box extends from the 25th to 75th percentiles, line in the middle of the box is the median and whiskers go down to the smallest value and up to the largest. **e**, Correlation plots of the serum levels of glucose vs. the gluconeogenic precursors, glycerol, lactate, pyruvate or alanine. Each dot corresponds to one serum sample. r, Two-tailed Pearson's correlation coefficient. Exact p values are reported in the Source Data file.

Supplementary Fig. 4

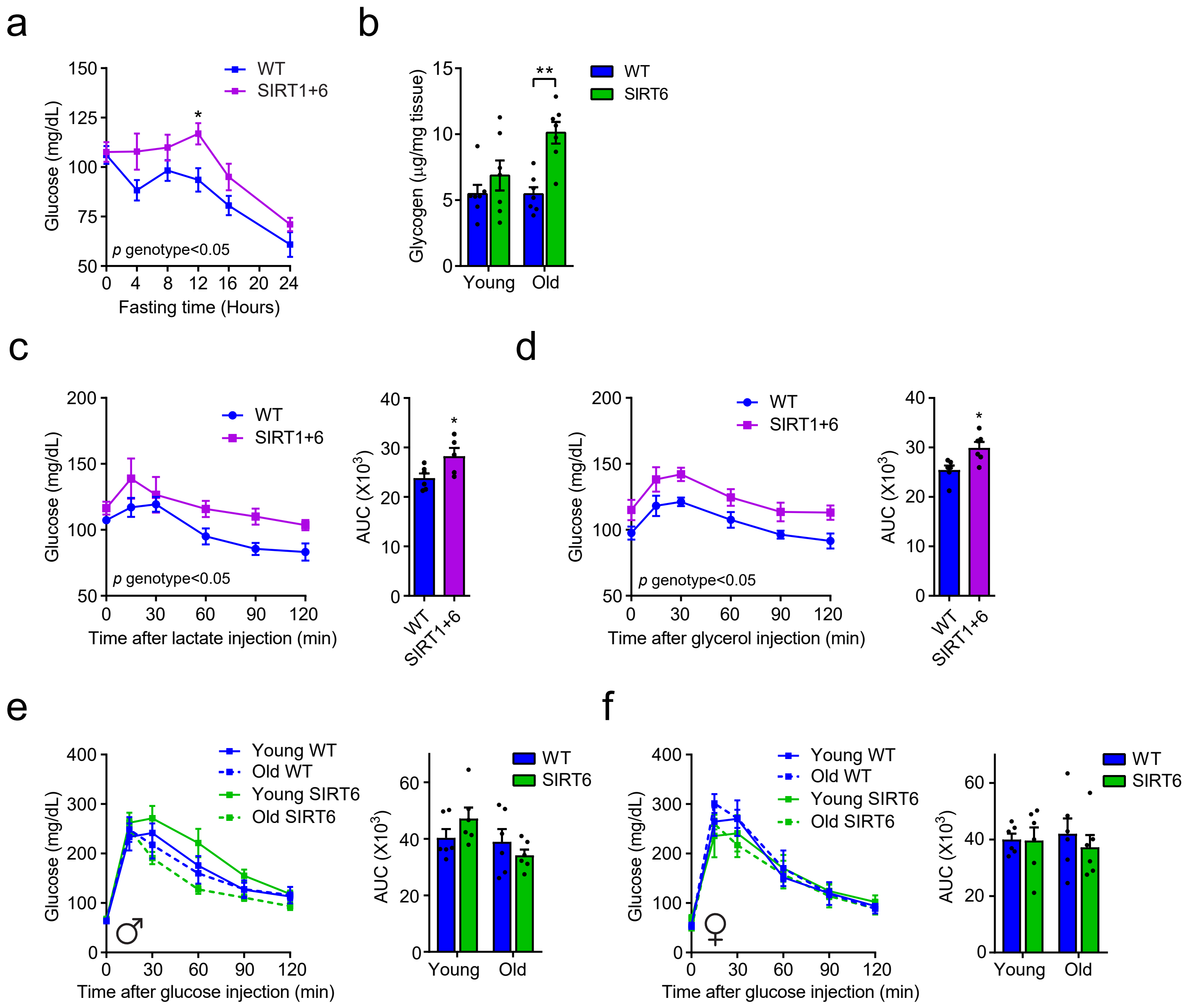

Supplementary Fig. 4. Extended tolerance tests and glycogen data. (Related to Fig. 3)

a, Blood glucose levels in 22 months old WT and SIRT1+6-tg mice at fed and fasted time points, as indicated in the graph. $n = 7$ WT mice, $n = 6$ SIRT1+6 mice, males. Two-way ANOVA with Sidak's post hoc. *, $p < 0.05$. **b**, Hepatic glycogen levels in 4h fasted young (6 months) and old (25 months) WT and SIRT6-tg male mice. $n = 7$ mice. Two-way ANOVA with Sidak's post hoc. **, $p < 0.01$. **c**, Lactate tolerance test in 24 months old 6h fasted male mice; $n = 6$ WT mice, $n = 5$ SIRT1+6 mice. **d**, Glycerol tolerance test in 24 months old 6h fasted male mice; $n = 6$ mice. The area under the curve (AUC) for each tolerance test in **c**,**d** is shown on the right. Two-way ANOVA for line graphs or student's t-test for AUC analysis. *, $p < 0.05$. For panels **a**,**c** and **d**, the values for old WT are the same as shown in Figs. **3a**,**c** and **d**, respectively, as SIRT1+6-tg mice were assayed in the same experiments. **e-f**, Glucose tolerance tests in young and old WT and SIRT6-tg mice of both sexes. $n = 6$ mice per group, at ages of 7 and 23 months for males, and 7 and 20 months for females. Statistical significance was calculated using three-way ANOVA for line graphs and two-way ANOVA with Fisher's LSD method for AUC. In all panels, values are mean ± SEM. Exact p values are reported in the Source Data file.

Supplementary Fig. 5

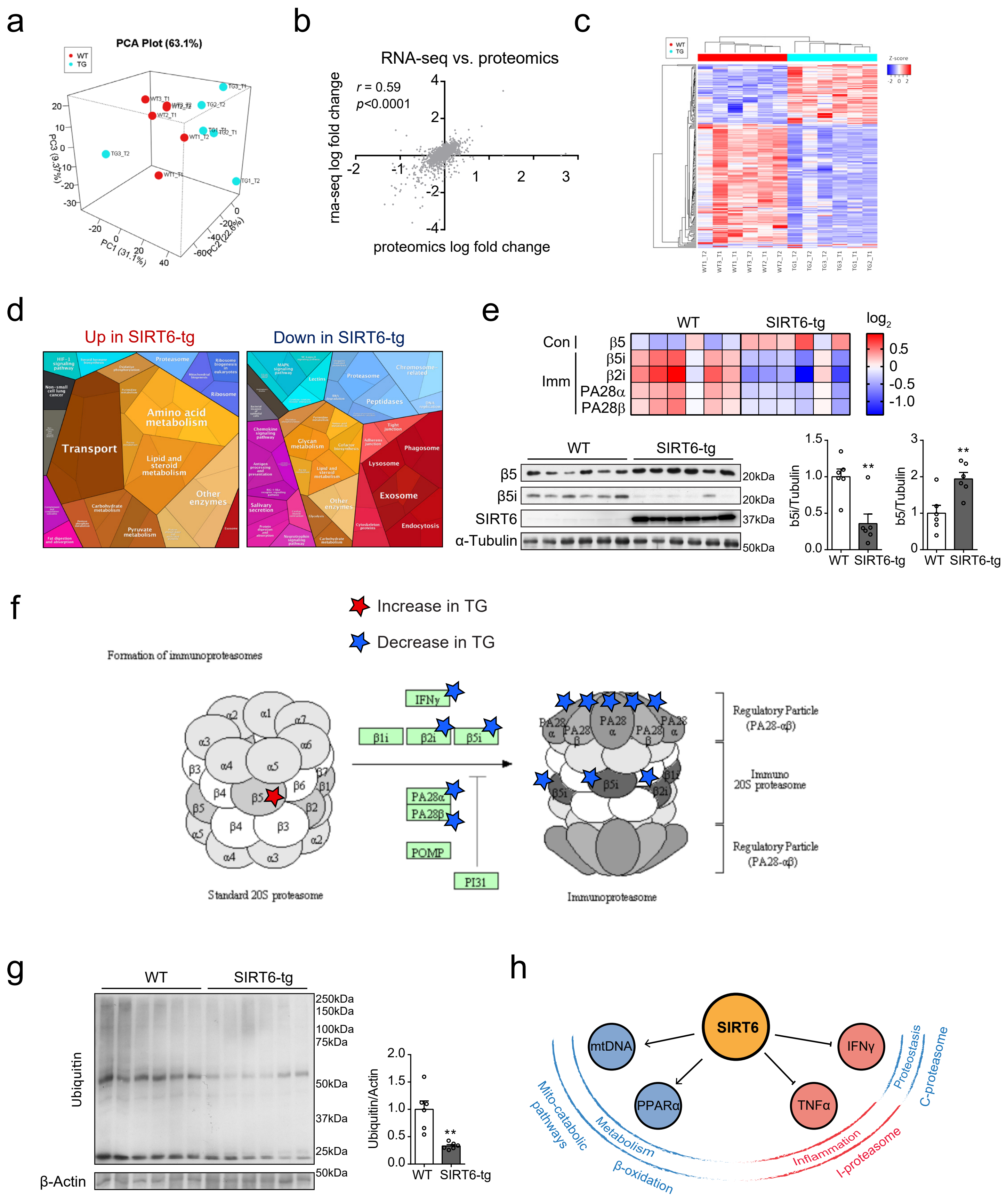

Supplementary Fig. 5. Liver proteomics. (Related to Fig. 4)

a, PCA of all 2785 identified proteins. **b**, Correlation between 2659 genes identified in both RNA-seq and proteomic analyses. Pearson r and p value (two-tailed) show significant positive correlation. **c**, Expression profile of the 185 differentially expressed proteins. **d**, Pathways up-regulated (left) and down-regulated (right) in SIRT6-tg. Each protein is shown by a polygon, and functionally related proteins are arranged in common regions (e.g. brown-yellow and purple represent metabolism and immune system, respectively). Polygon areas represent pathway abundance. **e**, Top: Heatmap generated from proteomic data show increased expression of constitutive proteasome β5 subunit and lower expression of immunoproteasome assembly proteins. Data are expressed by log2(fold change). Con, constitutive proteasome; Imm, immunoproteasome. Below: validations of β5 and β5i expression by western blot and corresponding quantification using ImageJ software. n = 6 mice per genotype. Values are mean ± SEM. **, $p < 0.01$, two-tailed student's t-test. **f**, Immunoproteasome model of KEGG proteasome pathway map depicting the proteins in this pathway that were significantly up-regulated or down-regulated in SIRT6-tg mice. **g**, Liver lysates from WT and SIRT6-tg mice were blotted against anti-ubiquitin antibody. The intensities of normalized protein expression, as determined by densitometry, are shown on the right. n = 6 mice per genotype. Values are mean ± SEM. **, $p < 0.01$, two-tailed student's t-test. Exact p values for **e**, **g**, are reported in the Source Data file. **h**, Scheme showing inflammatory and metabolic effects of SIRT6 in young liver. Blue, nodes activated by SIRT6; Red, nodes inhibited by SIRT6.

Supplementary Fig. 6

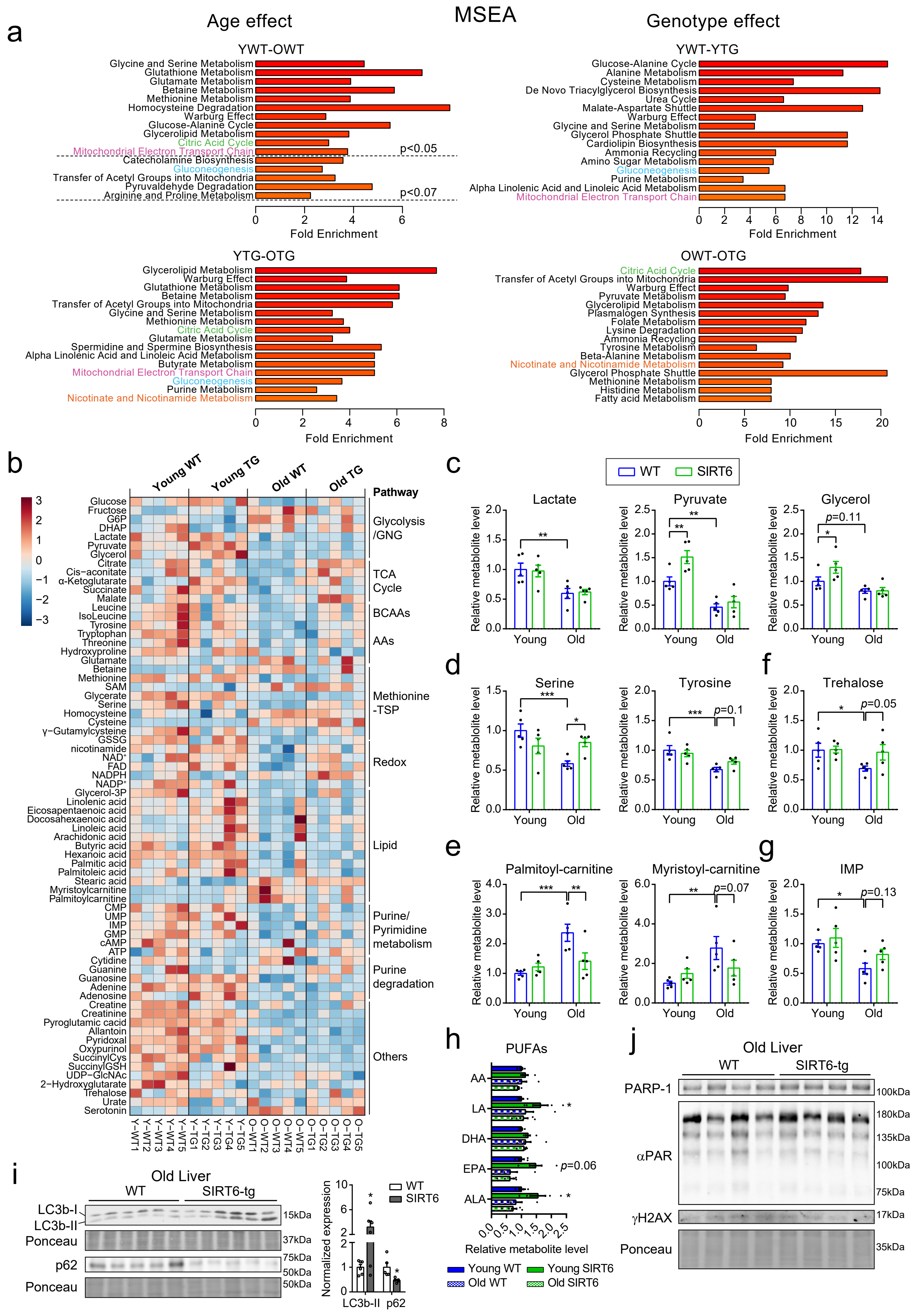

Supplementary Fig. 6. Liver metabolomics. (Related to Fig. 5)

a, Metabolite set enrichment analysis (MSEA) was performed using the list of statistically significant metabolites in each of the four comparisons presented in WT and TG mouse livers. Shown are the 16 top significant differential sets in each comparison. Several sets that were mutually affected by aging and genotype are shown in colored text. **b**, Heatmap showing the 70 metabolites that were significantly changed between groups, organized based on their metabolic pathway. Each square represents an individual mouse. TSP, Transsulfuration pathway. **c-h**, Metabolite levels of (**c**) hepatic GNG precursors, (**d**) amino acids, (**e**) fatty acyl-carnitines, (**f**) trehalose, (**g**) IMP, and (**h**) polyunsaturated fatty acids (PUFAs). $n = 5$ mice, analyzed by two-way ANOVA with Fisher's LSD method. ALA, Linolenic acid; EPA, Eicosapentaenoic acid; DHA, Docosahexaenoic acid; LA, Linoleic acid; AA, Arachidonic acid. **i**, Protein levels of LC3b ($n = 6$ mice) and p62 ($n = 5$ mice) in livers of old WT and SIRT6-tg littermates. ImageJ quantification normalized to ponceau is shown in the right, analyzed by two-tailed student's t-test. **j**, PARP1 protein levels, global PARylation and gamma-H2AX levels in livers of old WT and SIRT6-tg littermates. This experiment was repeated twice, independently, with similar results. $n = 4$ mice per genotype. *, $p < 0.05$, **, $p < 0.01$, ***, $p < 0.001$. In **c-i** values are mean ± SEM. For all panels, exact p values are reported in the Source Data file.

Supplementary Fig. 7

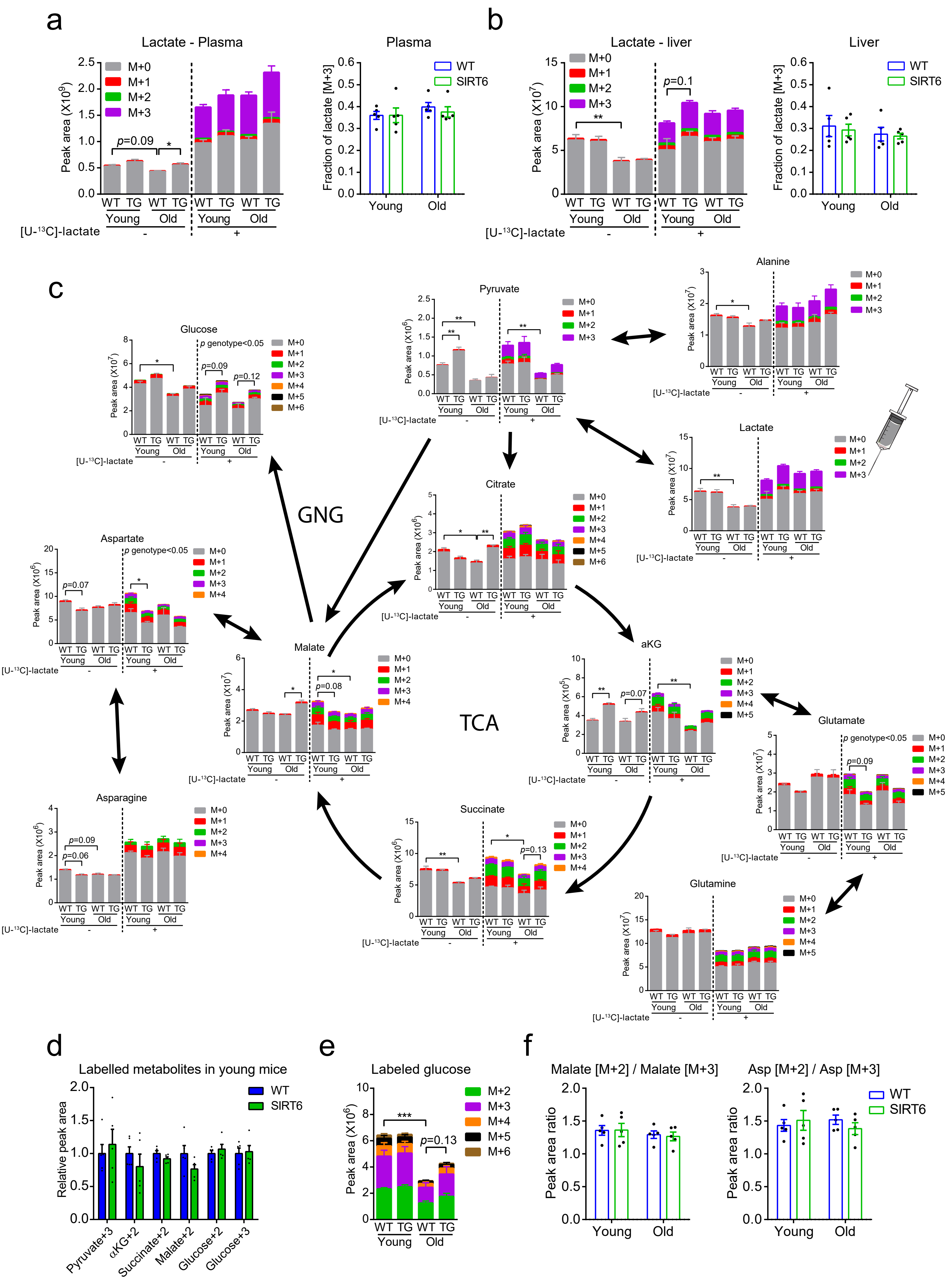

Supplementary Fig. 7. Lactate tracing. (Related to Fig. 6)

Liver metabolite levels in 6h fasted WT and TG mice, without lactate injection or 15 min after [U-^{13}C]-lactate injection. **a-b**, Plasma (**a**) and liver (**b**) lactate isotopologue abundances. Fractional abundances of lactate [M+3] after injection are shown to the right of each panel. **c**, Total levels and isotopologue abundances of liver TCA cycle and GNG-related metabolites. **d**, Relative abundances of hepatic labeled metabolites after [U-^{13}C]-lactate injection in young mice. **e**, Hepatic glucose M+2 to M+6 isotopologues after [U-^{13}C]-lactate injection. **f**, Ratios of hepatic M+2/M+3 malate and aspartate (Asp). For all panels, *n*=5 mice per group. Data were analyzed by two-way ANOVA with Fisher's LSD method for **a-c** and **e, f**, and by two-tailed student's t-test for **d**. *, $p < 0.05$, **, $p < 0.01$, ***, $p < 0.001$. In all panels, values are mean ± SEM. Exact p values for **e**, **f**, are reported in the Source Data file.

Supplementary Fig. 8

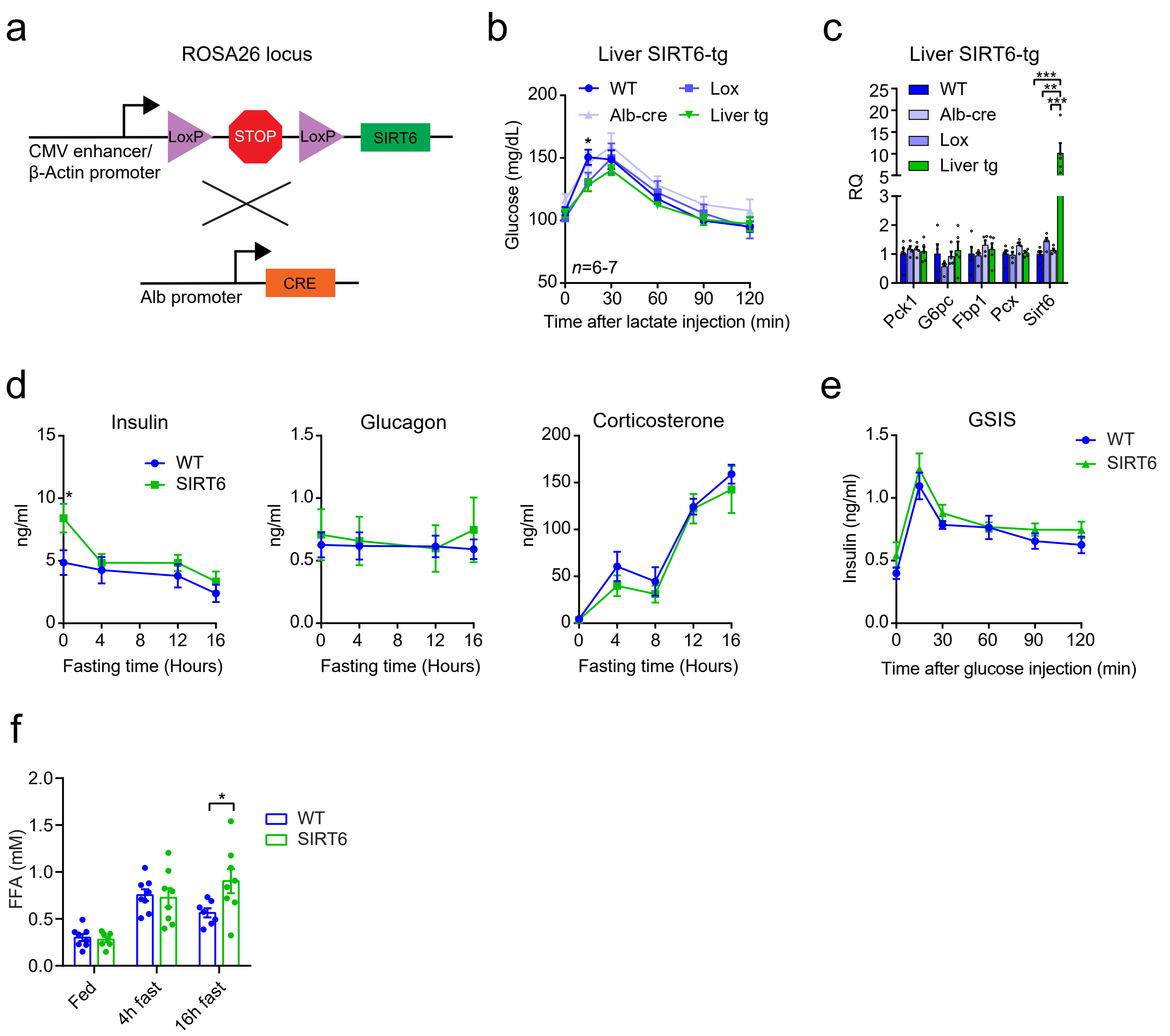

Supplementary Fig. 8. Extended liver-specific SIRT6-tg and hormonal profiling data. (Related to Fig. 7)

a, Liver-specific SIRT6-tg mice were derived by crossing mice containing Cre-recombinase induced by the liver specific albumin (Alb) promoter to mice containing a cassette of CMV enhancer/β-Actin promoter followed by lox-stop-lox and the SIRT6 coding sequence, integrated to the ROSA26 locus (SIRT6-Knock-in mice). **b**, Lactate tolerance test in liver-specific SIRT6-tg and appropriate littermate controls males at age of 7-9 months. Values are mean ± SEM of *n* = 6 WT, lox and Alb-cre mice, and *n* = 7 liver SIRT6-tg mice. Two-way ANOVA with Dunnett's post hoc. *, p<0.05 WT vs liver SIRT6 OE. Bars represent mean ± SEM. **c**, Expression of GNG-related genes in livers of 23-25 months old liver-specific SIRT6-tg and control littermates. SIRT6 expression is shown in the right. *n* = 5 WT and liver SIRT6-tg mice, *n* = 4 lox and Alb-cre mice, males. One-way ANOVA with Dunnett's post-hoc test. **, p < 0.01, ***, p < 0.001. **d**, Concentrations of insulin, glucagon and corticosterone in the serum of WT and whole body SIRT6-tg mice. For insulin and glucagon, *n* = 6 WT mice and *n* = 5 SIRT6-tg mice. For corticosterone, *n* = 5 mice. Males, 22 months. **e**, Glucose stimulated insulin secretion (GSIS) in whole body SIRT6-tg mice and WT littermates. *n* = 6 mice. Males, 23 months. **f**, Serum free fatty acids levels in 19-23 months old WT and SIRT6-tg littermates. *n* = 8 mice, males. For **d-f**, data were analyzed with two-way ANOVA (time, genotype) with Sidak's post-hoc test; *, p < 0.05. In **b-f**, values are mean ± SEM. Exact p values are reported in the Source Data file.
