# Supplementary material for: Restoration of energy homeostasis by SIRT6 extends healthy lifespan
Source: Nat Commun. 2021 May 28;12:3208. doi: 10.1038/s41467-021-23545-7 (PMC8163764; doi:10.1038/s41467-021-23545-7)
Supplement: Supplementary file 2 — Reporting summary [file 41467_2021_23545_MOESM2_ESM.pdf]

nature research

Corresponding author(s): Haim Y. Cohen

Last updated by author(s): Mar 15, 2021

Reporting Summary

Nature Research wishes to improve the reproducibility of the work that we publish. This form provides structure for consistency and transparency in reporting. For further information on Nature Research policies, see our Editorial Policies and the Editorial Policy Checklist.

Statistics

For all statistical analyses, confirm that the following items are present in the figure legend, table legend, main text, or Methods section.

n/a	Confirmed	
☐	☒	The exact sample size (*n*) for each experimental group/condition, given as a discrete number and unit of measurement
☐	☒	A statement on whether measurements were taken from distinct samples or whether the same sample was measured repeatedly
☐	☒	The statistical test(s) used AND whether they are one- or two-sided<br>*Only common tests should be described solely by name; describe more complex techniques in the Methods section.*
☐	☒	A description of all covariates tested
☐	☒	A description of any assumptions or corrections, such as tests of normality and adjustment for multiple comparisons
☐	☒	A full description of the statistical parameters including central tendency (e.g. means) or other basic estimates (e.g. regression coefficient) AND variation (e.g. standard deviation) or associated estimates of uncertainty (e.g. confidence intervals)
☐	☒	For null hypothesis testing, the test statistic (e.g. *F*, *t*, *r*) with confidence intervals, effect sizes, degrees of freedom and *P* value noted<br>*Give P values as exact values whenever suitable.*
☒	☐	For Bayesian analysis, information on the choice of priors and Markov chain Monte Carlo settings
☒	☐	For hierarchical and complex designs, identification of the appropriate level for tests and full reporting of outcomes
☒	☐	Estimates of effect sizes (e.g. Cohen's *d*, Pearson's *r*), indicating how they were calculated

Our web collection on statistics for biologists contains articles on many of the points above.

Software and code

Policy information about availability of computer code

Data collection

No software was used. We collected the data based on experiments done with our mice and tissues

Data analysis

RNA-seq:
STAR RNA-seq aligner software (version STAR_2.5.0a)
HTSeq-count version 0.6.1
DESeq2 version 1.16.1
Ingenuity Pathway Analysis - Fall Release (September 2017)
Gene Set Enrichment Analysis (GSEA, Broad Institute) – version 3.0
Proteomics:
MSConvert software (ProteoWizard 3.0.6002)
Mascot 2.4.1
X!Tandem CYCLONE (2010.12.01.1)
Uniprot database (version year 2016, appended with 115 known contaminant proteins)
Scaffold Q+ 4.4.6
R programming language 3.4.0
Serum metabolomics:
ChromaTOF version 2.32
Liver and plasma metabolite measurements:
Thermo Xcalibur 4.0
Compound Discoverer 3.0
MetaboAnalyst 4.0
TraceFinder 4.1

Western blot analysis:
ImageJ 1.52a
Plotting and Statistics:
GraphPad Prism ver 6.0 and 7.0
SPSS ver 20

For manuscripts utilizing custom algorithms or software that are central to the research but not yet described in published literature, software must be made available to editors and reviewers. We strongly encourage code deposition in a community repository (e.g. GitHub). See the Nature Research guidelines for submitting code & software for further information.

Data

Policy information about availability of data

All manuscripts must include a data availability statement. This statement should provide the following information, where applicable:

- Accession codes, unique identifiers, or web links for publicly available datasets
- A list of figures that have associated raw data
- A description of any restrictions on data availability

The source data underlying all box plots, bar and line graphs can be found in the online Source Data File, as well as the original uncropped western blots. Raw and processed RNA-seq data datasets were deposited to NCBI's GEO database under the accession number: GSE157838, the data can be accessed using the link https://www.ncbi.nlm.nih.gov/geo/query/acc.cgi?acc=GSE157838. . The mass spectrometry proteomics data have been deposited to the ProteomeXchange Consortium via the PRIDE90 partner repository with the dataset identifier PXD021447, and can be accessed using the link https://www.ebi.ac.uk/pride/archive/projects/PXD021447. . All raw metabolomics mass spectrometry data were deposited to NIH Common Fund's National Metabolomics Data Repository (NMDR) website, the Metabolomics Workbench, https://www.metabolomicsworkbench.org where it has been assigned Project IDs PR001005 (serum metabolomics) and PR001017 (liver metabolomics). The data can be accessed directly via it's Project DOIs https://doi.org/10.21228/M8SH6G and https://doi.org/10.21228/M87M4S. Databases used in this study: Uniprot (https://www.uniprot.org/), GO (http://geneontology.org/) and KEGG database (https://www.genome.jp/kegg/).

Field-specific reporting

Please select the one below that is the best fit for your research. If you are not sure, read the appropriate sections before making your selection.

☒ Life sciences ☐ Behavioural & social sciences ☐ Ecological, evolutionary & environmental sciences

For a reference copy of the document with all sections, see nature.com/documents/nr-reporting-summary-flat.pdf

Life sciences study design

All studies must disclose on these points even when the disclosure is negative.

Sample size	For lifespan studies, sample size selection was based on literature (https://doi.org/10.1002/9780470942390.mo140195) and the lab's previous experience (https://doi.org/10.1038/nature10815) that using 40 mice of each sex on average is sufficient to detect a 20% change in lifespan in α=0.05 and 80% power. For other in vivo experiments, sample size selection was based on the extensive in vivo experience in the lab. For our outcome variables, we usually observed standard deviation of around 20%, and therefore average of 6 mice per group is sufficient to detect 33% change with 80% power.
Data exclusions	Grubbs test was used to detect statistically significant outliers
Replication	For all experiments we used multiple biological replicates, as indicated in the figure legends. The data represents experiments performed using multiple mouse cohorts with different birth dates. All attempts for replication were successful.
Randomization	Allocation was random
Blinding	Investigators were blinded to group allocation during data collection and data analyses.

Reporting for specific materials, systems and methods

We require information from authors about some types of materials, experimental systems and methods used in many studies. Here, indicate whether each material, system or method listed is relevant to your study. If you are not sure if a list item applies to your research, read the appropriate section before selecting a response.

Materials & experimental systems

n/a	Involved in the study	
	☒	Antibodies
☒	☐	Eukaryotic cell lines
☒	☐	Palaeontology and archaeology
	☒	Animals and other organisms
☒	☐	Human research participants
☒	☐	Clinical data
☒	☐	Dual use research of concern

Methods

n/a	Involved in the study	
☒	☐	ChIP-seq
☒	☐	Flow cytometry
☒	☐	MRI-based neuroimaging

Antibodies

Antibodies used

We used commercial primary antibodies directed against β-actin (C4, sc-47778, at 1:1000 dilution), Ub (P4D1, sc-8017, 1:1000), PARP1 (F-2, sc-8007, 1:1000), α-tubulin (Sigma Aldrich clone B-5-1-2, T5168, 1:7000), SIRT6 (D8D12, cst-12486, 1:1000), PSMB5 (β5) (D1H6B, cst-12919, 1:1000), PSMB8 (β5i) (D1K7X, cst-13635, 1:1000), LC3B (cst-2775, 1:1000), p62 (cst-5114, 1:1000), HSL (D6W5S, cst-18381, 1:1000), phospho-HSL (ser 563) (cst-4139, 1:1000), phospho-Histone H2A.X (Ser139) (MilliporeSigma clone JBW301, 05-636, 1:200), PAR (Trevigen, 4335-AMC-050, 1:1000), NMNAT1 (ab45652, 1:1000) and TDO2 (LSBio, LS-C748245, 1:500). Anti-mouse or anti-rabbit HRP-conjugated antibodies (Jackson ImmunoResearch, 115-035-146 and 111-035-003, 1:15000) were used as secondary antibodies.

Validation

All antibodies used in this study were commercially developed and validated by the companies as follows: β-actin and Ub Abs were validated by Santa Cruz Biotechnology using transfection of the targeted protein. PARP-1 Ab was validated by Santa Cruz Biotechnology using siRNA against the targeted protein. α-tubulin Ab was validated by Sigma Aldrich using Independent Antibody Verification, which demonstrates antibody specificity using multiple antibodies against target in IHC or ICC. SIRT6 Ab was validated by Cell Signaling Technology using shRNA and KO cells. PSMB5, PSMB8 and LC3B were validated by Cell Signaling Technology by transfection of the target protein and chemical treatment that induces/inhibits expression. p62 was validated by Cell Signaling Technology by transfection of the target protein. HSL and p-HSL were validated by Cell Signaling Technology by measuring antibody signal in model systems with known presence/absence of target signal. For Cell Signaling Technology antibodies, species reactivity is determined by testing in at least one approved application. phospho-Histone H2A.X (Ser139) was validated by MilliporeSigma using chemical treatment that induces its expression. For PAR, Trevigen have provided references for publications using this antibody and showing its specificity. NMNAT1 was validated by Abcam using human skeletal muscle tissue lysate. TDO2 was validated by LSBio using various cell lines and mouse and rat tissue lysates.

Animals and other organisms

Policy information about studies involving animals; ARRIVE guidelines recommended for reporting animal research

Laboratory animals

C57BL/6JOlaHsd WT mice, SIRT6-tg mice, SIRT1-tg mice, SIRT1+6-tg mice, age 3-30 months, males and females. Alb-cre mice (Jax Stock No: 003574), lox-stop-lox SIRT6-Knockin mice, liver SIRT6-tg mice, age 3-25 months, males.

Wild animals

None

Field-collected samples

None

Ethics oversight

Bar-Ilan Institutional Animal Care and Use Committee.

Note that full information on the approval of the study protocol must also be provided in the manuscript.
